# Predicting the impact of outdoor vector control interventions on malaria transmission intensity from semi-field studies

**DOI:** 10.1186/s13071-020-04560-x

**Published:** 2021-01-20

**Authors:** Adrian Denz, Margaret M. Njoroge, Mgeni M. Tambwe, Clara Champagne, Fredros Okumu, Joop J. A. van Loon, Alexandra Hiscox, Adam Saddler, Ulrike Fillinger, Sarah J. Moore, Nakul Chitnis

**Affiliations:** 1 Department of Epidemiology and Public Health, Swiss Tropical and Public Health Institute, Socinstrasse 57, 4051 Basel, Switzerland; 2 University of Basel, Petersplatz 1, Basel, Switzerland; 3 Human Health Theme, International Centre of Insect Physiology and Ecology (icipe), 00100 Nairobi, Kenya; 4 Laboratory of Entomology, Wageningen University and Research, P.O. Box 16, 6700 AA Wageningen, The Netherlands; 5 Environmental Health and Ecological Sciences Department, Ifakara Health Institute, P.O. Box 53, Ifakara, Tanzania; 6 ARCTEC, London School of Hygiene and Tropical Medicine, Keppel Street, WC1E 7HT London, UK; 7 School of Public Health, Faculty of Health Science, University of the Witwatersrand, Johannesburg, South Africa; 8 School of Life Science and Biotechnology, Nelson Mandela African Institution of Science and Technology, P.O. Box 447, Arusha, Tanzania; 9 Institute of Biodiversity, Animal Health and Comparative Medicine, University of Glasgow, Glasgow, UK

**Keywords:** Malaria, *Anopheles arabiensis*, Vector control, Outdoor transmission, Spatial repellent, Volatile pyrethroids, Semi-field experiments, Community-level impact, Stochastic modelling, Hierarchical Bayesian model

## Abstract

**Background:**

Semi-field experiments with human landing catch (HLC) measure as the outcome are an important step in the development of novel vector control interventions against outdoor transmission of malaria since they provide good estimates of personal protection. However, it is often infeasible to determine whether the reduction in HLC counts is due to mosquito mortality or repellency, especially considering that spatial repellents based on volatile pyrethroids might induce both. Due to the vastly different impact of repellency and mortality on transmission, the community-level impact of spatial repellents can not be estimated from such semi-field experiments.

**Methods:**

We present a new stochastic model that is able to estimate for any product inhibiting outdoor biting, its repelling effect* versus* its killing and disarming (preventing host-seeking until the next night) effects, based only on time-stratified HLC data from controlled semi-field experiments. For parameter inference, a Bayesian hierarchical model is used to account for nightly variation of semi-field experimental conditions. We estimate the impact of the products on the vectorial capacity of the given *Anopheles* species using an existing mathematical model. With this methodology, we analysed data from recent semi-field studies in Kenya and Tanzania on the impact of transfluthrin-treated eave ribbons, the odour-baited Suna trap and their combination (push–pull system) on HLC of *Anopheles arabiensis* in the peridomestic area.

**Results:**

Complementing previous analyses of personal protection, we found that the transfluthrin-treated eave ribbons act mainly by killing or disarming mosquitoes. Depending on the actual ratio of disarming * versus* killing, the vectorial capacity of *An. arabiensis* is reduced by 41 to 96% at 70% coverage with the transfluthrin-treated eave ribbons and by 38 to 82% at the same coverage with the push–pull system, under the assumption of a similar impact on biting indoors compared to outdoors.

**Conclusions:**

The results of this analysis of semi-field data suggest that transfluthrin-treated eave ribbons are a promising tool against malaria transmission by *An. arabiensis* in the peridomestic area, since they provide both personal and community protection. Our modelling framework can estimate the community-level impact of any tool intervening during the mosquito host-seeking state using data from only semi-field experiments with time-stratified HLC.
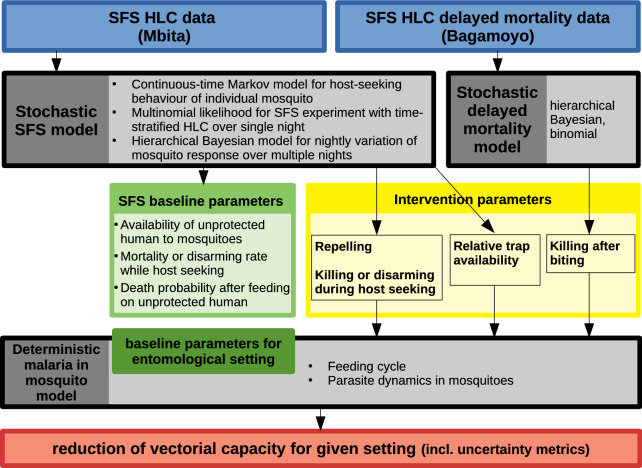

## Introduction

Although malaria transmission has decreased substantially since 2000, primarily due to the use of insecticide-treated nets (ITNs), the incidence of clinical cases has plateaued in the last few years. Despite room for improvement in coverage, it is evident that residual transmission will persist since not all malaria transmission can be stopped by ITNs and indoor residual spraying (IRS) [2]. New vector control tools against residual transmission need to be invented, developed, evaluated and selected [3]. There is growing evidence that a substantial part of residual malaria transmission occurs outdoors [4–7] and in the evening as well as mornings [8]. A large focus of human outdoor activities during mosquito biting times is the peridomestic area [9, 10], which we define as the space near houses, usually within 10 m, where household members spend time before going indoors to sleep. Spatial repellents are a promising tool to target the peridomestic area and other confined outdoor spaces, especially in view of costs and minimal requirement of user compliance [11]. Volatile pyrethroids impregnated into hessian fabric [12–14] and recently applied as eave ribbons [15, 16] have shown particular promise.

Depending on the active ingredients and the dosage of spatial repellents, different modes of action are known [17, 18], with very different impacts on malaria transmission. We categorise the modes of action into the following effects:

- Repellency, here defined as inhibiting landing on a protected host but otherwise not affecting the mosquito and potentially increasing biting of unprotected hosts, which provides only personal protection;
- Disarming, here defined as inhibiting host-seeking behaviour until the next night without killing the mosquito, which provides both personal and community protection;
- Killing before biting, which provides both personal and community protection; and
- Killing after biting, which provides only community protection.

An important drawback of spatial repellents with a repelling effect is that mosquitoes might be pushed towards unprotected hosts, thereby increasing their biting risk [19]. Therefore, spatial repellents may be combined with odour-baited traps [20] to form a push–pull system, with the goal of diverting mosquitoes to a trap to kill them instead of diverting them to another host. Earlier studies found a significant reduction of house-entry by push–pull systems compared to push–only systems [21, 22], while studies focusing on outdoor biting found that the push–pull system was only marginally more effective than [23] or equally effective [24] as the corresponding push formulations in reducing outdoor transmission. Recently, a large semi-field study in Kenya and Tanzania identified and tested candidate spatial repellents, odour-baited traps and combined push–pull systems targeting outdoor transmission in the peridomestic area. The Kenyan data showed a strong reduction of outdoor HLC counts by the transfluthrin-treated eave-ribbons, a more moderate reduction by the push–pull system and no effect of the Suna trap baited with the human odour mimic ‘Mbita blend 5’ (MB5) and carbon dioxide [16].

Semi-field experiments in screen houses are an important step in the product development of spatial repellents and other tools targeting outdoor transmission. They allow experiments to be conducted with freely flying insectary-reared mosquitoes that are disease free and safe to human volunteers conducting human landing catches (HLC). Such experiments provide good estimates of user-level impact* via* the relative reduction of HLC counts (‘protective efficacy’). Unfortunately, it is difficult to distinguish different modes of action in such semi-field experiments, since all modes manifest in a reduction of HLC counts while it is often not feasible to recapture the mosquitoes—dead or alive—that were not caught by HLC. However, to quantify the community-level impact of a given product on malaria transmission it is necessary to know its effect(s) in terms of repellency, disarming and killing both before and after biting. In an extreme case, it may happen that in a semi-field experiment a tool X with high repelling but low killing effect shows higher protective efficacy than a tool Y with high killing effect, while in the field, tool Y would reduce outdoor transmission much more than tool X.

Here, we present a new stochastic model for semi-field experiments with HLC that is able to estimate the differentiated effect of spatial repellents in terms of repellency on one hand, and disarming or killing on the other hand, based on time-stratified HLC data only. This mechanistic modelling approach is needed because a purely statistical model applied to the semi-field data would only estimate the reduction of biting on the user of the intervention during 12 h and fail to accurately estimate the effect of the intervention on vectorial capacity, which also includes the community effect due to the disarming and the increased mortality of the mosquito population. Odour-baited traps are included in the model to allow for analysis of push–pull experiments. This stochastic model is based on parts of the deterministic ‘malaria in mosquito’ model [25], which is then used to predict the community-level impact (vectorial capacity) of the tested tool based on the differentiated effect estimates obtained from the stochastic model. Hierarchical Bayesian modelling is used to account for night-specific variability, and credible intervals are used to quantify estimation uncertainty. For dengue vector control, a similar modelling approach was pursued by ten Bosch et al. [26] to predict the effect on transmission of spatial repellents tested in semi-field experiments, with the advantage that the experimental design focusing on house entry of *Aedes* mosquitoes allowed for direct measures of mortality during the host-seeking state.

We use this model, to predict the community-level impact of insecticide-treated eave ribbons, the Suna trap and their combination (push–pull) by incorporating both bite prevention and mosquitocidal effects estimated from recent semi-field studies in Kenya and Tanzania [16]. This complements the analysis carried out in that study [16] which focused on user-level impact. With this modelling framework we hope to help accelerate product development by providing estimates of community-level impact in the early stage of semi-field experiments.

## Methods

### Semi-field experiments

We briefly describe the semi-field experiments with time-stratified HLC as conducted in Kenya and Tanzania in order to assess spatial repellents (push), odour-baited traps (pull) and their combination (push–pull); for more details see [16].

#### Interventions

The spatial repellent consisted of hessian strips (roughly woven sisal fabric) impregnated with 2.5 g transfluthrin per square meter as described in [12, 13] and placed at the eaves of the experimental hut as described in [15]. Throughout this article we refer to this specific intervention as the ‘spatial repellent’, denoted as ‘R’. The odour-baited trap consisted of the Suna trap [27], baited with a cartridge containing the human odour mimic MB5 [28, 29] and with carbon dioxide produced by yeast and molasses fermentation [30]. Throughout this article we refer to this specific intervention as ‘trap’, denoted with ‘T’. The combination of trap (‘pull’) and spatial repellent (‘push’) is called a ‘push–pull’ system, referred to with the letter ‘P’.

#### Experimental sites

The semi-field experiments were conducted at the International Centre of Insect Physiology and Ecology (icipe) campus in Mbita, Kenya, and at the Ifakara Health Institute (IHI) campus in Bagamoyo, Tanzania. Mbita (0°41′N, 34°19′E; 1150 m a.s.l.) lies in western Kenya at the shores of Lake Victoria, and Bagamoyo (6°26′S, 38°54′E; 8 m a.s.l.) lies on the Tanzanian coast. Parallel treatment and control experiments were carried out at two large semi-field sites (screen houses; 27 × 11 m, 4.4 m high) in each location. The ground of the screen houses was cleared of vegetation to avoid sugar feeding, and spiders were removed. Inside the screen house an unoccupied experimental hut was positioned at about one-third of the length of the screen house. The volunteer conducting HLC was positioned 2.5 m from the hut and the trap positioned 5 m from the hut, both towards the center of the screen house.

#### Mosquitoes

All experiments were conducted with the Mbita strain of *Anopheles arabiensis* mosquitoes, which shows moderate resistance to pyrethroids (around 92% mortality in the World Health Organisation cone bioassays). Mosquitoes used in the experiments were reared in insectaries, were aged 3–5 days, never had a blood meal prior to the experiment and were starved prior to the experiment.

#### Experimental design

All interventions—spatial repellent (R), trap (T) and push–pull (P)—were tested in 16 replicates (nights), each with its own control consisting of the same device with active ingredients removed. The experiments were fully blinded and volunteer as well as screen house were randomised. At the start of the experiment at 7:00 pm, a total of 160 *An. arabiensis* mosquitoes are released from four cups placed at the corners of the screen house. The release number was later corrected by the number of mosquitoes (dead or alive) found in the release cup at the end of the experiment. Time-stratified HLC was conducted during hours 0–1, 1–2 and 2–3, as counted from the start of the experiment, as well as again during hours 11.5–12 the following morning in order to remove all remaining mosquitoes from the screen house. For the trap experiments, the number of trap catches was evaluated after 3 and 12 h, while for the push–pull experiments trap catches were only measured after 3 h. When the cumulative HLC count in the control experiment was less than half of the release number, then all experiments of that night were discarded and repeated. For the experiments run in Bagamoyo, HLC was conducted during hours 0–1, 1–2, 2–3 and 3–4, and the mosquitoes caught with HLC were kept in the insectary for 12 h after the end of the semi-field experiment in order to measure delayed mortality. An example of the collected data for one replicate of push–pull with control is shown in Table 1.

**Table 1:** Example of semi-field data for one night

Experimental setting	*n*	$x_{\text {H1}}$	$x_{\text {H2}}$	$x_{\text {H3}}$	$x_{\text {H4}}$	$x_{\text {T}}$	$x_{\text {L}}$
Control	158	52	15	14	1	0	70
Intervention	145	21	10	12	8	1	93

HLC, Human landing catch

*n* denotes the number of mosquitoes who left the release cups; $x_{\text {H1}}$, $x_{\text {H2}}$, $x_{\text {H3}}$ and $x_{\text {H4}}$ denote the number of mosquitoes caught in HLC periods 1, 2, 3 and 4, respectively; $x_{\text {T}}$ denotes the number of mosquitoes caught in the trap (T); $x_{\text {L}}$ denotes the number of mosquitoes lost to follow-up throughout the night

### Overview of modelling methodology

**Fig. 1:**
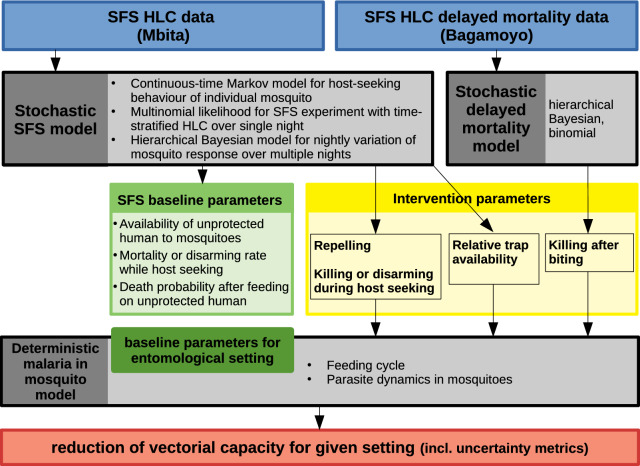
Overview of modelling methodology with the two novel models ‘stochastic SFS model’ and ‘stochastic delayed mortality model’

An overview of the modelling methodology is shown in Fig. 1, including an introduction to the existing deterministic ‘malaria in mosquito’ model.

#### Deterministic ‘malaria in mosquito’ model

We base our modelling framework on a deterministic, discrete-time model of the ‘dynamics of malaria in a mosquito population feeding on, infecting and getting infected from a heterogeneous population of hosts’ [25]. This model couples the mosquito feeding cycle with the dynamics of infections with malaria in mosquitoes and allows us to compute entomological endpoints, such as the vectorial capacity and the entomological inoculation rate (EIR), as a function of parameters describing the vector bionomics, the host population and the vector control interventions. The model [25] is linked to an individual-based stochastic model of malaria in humans in the simulation platform OpenMalaria [31, 32], which is not used here but which will prospectively allow us to predict effects on clinical incidence of malaria for the given interventions.

The mosquito feeding cycle in this deterministic model [25] consists of the five states listed in Table 2, each with a fixed duration.

**Table 2:** States of the mosquito feeding cycle in the deterministic model [25]

State	Description	Intervention parameterisation
*A*	Host seeking	Repelling effect ($\pi$), killing/disarming effect during host seeking state ($\kappa$), relative trap availability ($\rho$)
$B_{i}$	Encountered host of type *i*	–
$C_{i}$	Fed on host of type *i*	Postprandial killing effect ($\xi$)
$D_{i}$	Resting after having fed on host of type *i*	–
$E_{i}$	Ovipositing after having fed on host of type *i*	–

The index *i* represents specific host types; for example, ‘unprotected humans’ and ‘humans protected by some intervention’

The last column lists the parameters describing the effect of vector control interventions on the corresponding stage of the feeding cycle

**Fig. 2:**
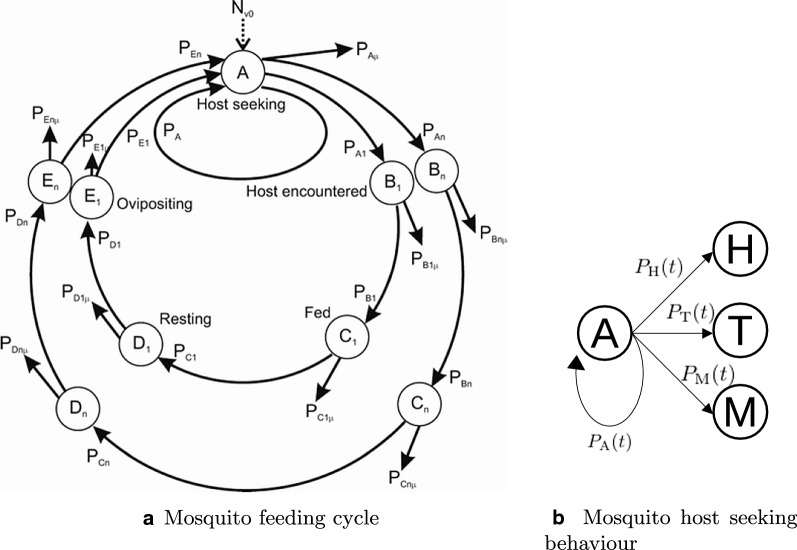
Schematics for mosquito feeding model. **a** The deterministic mosquito feeding cycle according to the model described in [25]. (Model is reproduced here with permission [25]; notations are provided in Table 2). Mosquitoes emerge from breeding sites and survive to the host-seeking state (*A*) at rate $N_{v0}$. From among all mosquitoes in the host-seeking state (*A*), a proportion $P_{A^{i}}$ encounter a host of type *i* ($B_{i}$). For all available host types indexed with *i*, a proportion $P_{A}$ stay in state *A* and a proportion $P_{A\mu }$ die. Depending on the host type encountered, the mosquitoes follow distinct cycles until they reach state *A* again or die. Of all mosquitoes in state $B_{i}$, a proportion $P_{B_{i}}$ successfully feed on a host of type *i* ($C_{i}$) and a proportion $P_{B_{i}\mu }$ die ($P_{B_{i}\mu } = 1 - P_{B_{i}}$). Of all mosquitoes in state $C_{i}$, a proportion $P_{C_{i}}$ move to the ‘resting state after having fed on a host of type *i*’ ($D_{i}$) and a proportion $P_{C_{i}\mu }$ die ($P_{C_{i}\mu } = 1 - P_{C_{i}}$ ). Of all mosquitoes in state $D_{i}$, a proportion $P_{D_{i}}$ move to the ‘ovipositing state after having fed on a host of type *i*’ ($E_{i}$) and a proportion $P_{D_{i}\mu }$ die ($P_{D_{i}\mu } = 1 - P_{D_{i}}$ ). Of all mosquitoes in state $E_{i}$, a proportion $P_{D_{i}}$ find a ovipositing site, lay eggs and return to the host-seeking state (*A*) and a proportion $P_{E_{i}\mu }$ die ($P_{E_{i}\mu } = 1 - P_{E_{i}}$ ).** b** Continuous-time Markov model for the behaviour of an individual mosquito in the host-seeking state (*A*) of a feeding cycle with three host types. A, H, T and M represent the states of host seeking, HLC, trap catch and death, respectively. $P_{\text {A}}(t), P_{\text {H}}(t), P_{\text {T}}(t)$ and $P_{\text {M}}(t)$ represent the probabilities to move within a time window of duration *t* from state A to state A, H, T and M, respectively. It is assumed that these probabilities are independent of how long the mosquito has already stayed in state A before the given time window (Markov property)

In each state, a proportion of the mosquitoes move to the next state and the rest die, with exception of state *A* of duration 1 day, which can be repeated multiple times. All mosquitoes in state *A* start host seeking at the beginning of the night and encounter a host with host type-specific availability rates or die at a constant per capita rate. Host types are indexed by *i* and could, for example, consist of humans protected and unprotected by a specific vector control intervention. We define ‘encountering’ as a commitment to either feed on a particular host type or die, and mosquitoes that are deterred but not killed are therefore considered to always have remained in the host-seeking state. If at the end of the night (of duration $\theta _{d}$), they have neither encountered a host nor died, they remain in the host-seeking state until the next night. For each host type there is a distinct cycle with distinct transition probabilities, in which a mosquito stays after encountering a host until it reaches state *A* again or dies, as illustrated in Fig. 2a. The model does not distinguish between indoor and outdoor biting explicitly, and represents the weighted average over both indoor and outdoor biting.

#### Modelling vector control interventions

Vector control interventions are modelled by introducing a separate host type for the hosts protected by the intervention, with transition probabilities altered according to the intervention effect. How transition probabilities are altered by interventions intervening in the host-seeking state is captured by the parameters listed in the last column of Table 2. The coverage level of a given intervention is implemented by the number of hosts of the host type protected by the intervention. In contrast to this, we implement traps by adding for each trap a dummy host that kills all mosquitoes after encountering, parameterised solely by the relative availability of a trap compared to an unprotected human host.

To incorporate the killing and disarming effects of an intervention we add for every protected human host a ’shadow host’ that is unable to contract and transmit malaria, equipped with an availability rate corresponding to the killing/disarming effect of the intervention. To model killing we set the proportion of mosquitoes that die after encountering a shadow host to 1. To model disarming (here defined as inhibiting host-seeking behaviour until the next night, without killing the mosquito) we add the proportion of mosquitoes encountering a shadow host to the proportion of mosquitoes staying in host-seeking state *A* and otherwise do not consider shadow hosts in the feeding cycle. Modelling inhibition of host-seeking behaviour for multiple days is addressed in Additional file 1: Appendix E.

To parameterise a given intervention from semi-field experimental data we developed two novel stochastic models: The stochastic semi-field system (SFS) model for HLC data corresponds to the deterministic continuous-time model for state *A* of the feeding cycle in the model [25] and is developed in four steps in the following section. Apart from intervention parameterisation, this model also provides estimates of baseline parameters of the semi-field system to better understand the dynamics of the semi-field experiments as such. All parameters of this model are fitted to data from the semi-field experiments conducted in Mbita. The stochastic delayed mortality model for delayed mortality in mosquitoes collected by HLC corresponds to the deterministic model for state *C* of the feeding cycle in the model [25] and is described in a separate section. All parameters of this model are fitted to data collected from Bagamoyo.

### Model for experiments in semi-field system with HLC data

#### Continuous-time Markov model for host-seeking behaviour of individual mosquitoes

We develop a stochastic process for the host-seeking behaviour of a single mosquito which corresponds to the deterministic ordinary differential equation (ODE) model for the population level dynamics in state *A* of the feeding cycle in [25]. A stochastic process model has the advantage that the stochasticity is built in the behaviour of each single mosquito, while a statistical model for fitting the existing ODE model to data would need to impose assumptions on the probability distribution of the data. The chosen continuous-time Markov chain model is the simplest stochastic process that recovers the existing ODE model in the mean. The notation agrees largely with the one in [25], but we omit state *B*, which is difficult to observe in semi-field experiments with outdoor HLC. The new notation is introduced in the main text and also displayed in Tables 3, 4 and 5.

**Table 3:** Notation for semi-field data and semi-field model

Symbol	Description	Unit
$\text {C}$	Wildcard for control arm of any experiment	
$\text {I}$	Wildcard for intervention arm of any experiment	
*n*	Number of mosquitoes released at the beginning of the experimental night	Dimensionless
$x_{\text {H}1}, x_{\text {H}2}, x_{\text {H}3}$ and $x_{\text {H}4}$	Number of mosquitoes caught in HLC period 1, 2, 3 and 4, respectively	Dimensionless
$x_{\text {T}}$	Number of mosquitoes caught in the trap at the end of the experiment	Dimensionless
$x_{\text {L}}$	Number of mosquitoes lost to follow-up at the end of the experiment	Dimensionless
*k*	Index for night $k \in \{1, \ldots , 16 \}$	
$D_{k}$, $D_{k}[\text {I}]$ or $D_{k}[\text {C}]$	Data $(x_{\text {H1}}, x_{\text {H2}}, x_{\text {H3}}, x_{\text {H4}}, x_{\text {T}}, x_{\text {L}})$ from night *k* of unspecified, control or intervention arm, respectively	Dimensionless vector
*D*, $D[\text {I}]$ or $D[\text {C}]$	Collection of $D_{k}$, $D_{k}[\text {I}]$ or $D_{k}[\text {C}]$ over all available nights *k* of respective arm	Dimensionless vector
*t*	Time	Hour
$P_{\text {A}}(t)$	Probability to stay in host seeking state ($\text {A}$) within time *t*	Probability
$P_{\text {H}}(t)$	Probability to get caught by HLC (move from $\text {A}$ to $\text {H}$) within time *t*	Probability
$P_{\text {T}}(t)$	Probability to get caught in the trap (move from $\text {A}$ to $\text {T}$) within time *t*	Probability
$P_{\text {M}}(t)$	Probability to die (move from $\text {A}$ to $\text {M}$) within time *t*; $P_{\text {M}}(\theta _{d})$ corresponds to $\text {P}_{\text {A}\mu }$ in Fig. 2a	Probability
$p_{\text {H1}}, p_{\text {H2}}, p_{\text {H3}}, p_{\text {H4}}$	Probability for a mosquito to get caught in HLC period 1, 2, 3 or 4, respectively	Probability
$p_{\text {T}}$	Probability for a mosquito to get caught in the trap	Probability
$p_{\text {L}}$	Probability for a mosquito to be lost to follow-up throughout the semi-field experiment, irrespective of being dead or alive.	Probability
$\text {pr}$	Logarithm of probability density function	Log-density

**Table 4:** Dependent parameters of semi-field model

Symbol	Description	Unit
$\alpha _{\text {H}_{k}}$	Human availability rate in night *k*	$\text {h}^{-1}$
$\alpha _{\text {T}_{k}}$	Trap availability rate in night *k*	$\text {h}^{-1}$
$\mu _{k}$	Mosquito mortality or disarming rate in night *k*	$\text {h}^{-1}$
$\alpha _{\text {H}}$	Mean human availability rate over all nights	$\text {h}^{-1}$
$\alpha _{\text {T}}$	Mean trap availabitrap availability rate acts as an additional lity rate over all nights	$\text {h}^{-1}$
$\mu$	Mean mosquito mortality or disarming rate over all nights	$\text {h}^{-1}$

Parameters may be equipped with $[\text {C}]$ or $[\text {I}]$ to denote a control or intervention arm, respectively, to which they are fitted

*k* is the index for a given night, as defined in Table 3

**Table 5:** Independent parameters of semi-field model

Symbol	Description	Unit	Prior
*a*	Mean of $\log (\alpha _{\text {H}_{k}})$	$\log (\text {h}^{-1})$	${\mathcal {N}}(0,6)$
*b*	Mean of $\log (\alpha _{\text {T}_{k}})$	$\log (\text {h}^{-1})$	${\mathcal {N}}(0,6)$
*m*	Mean of $\log (\mu _{k})$	$\log (\text {h}^{-1})$	${\mathcal {N}}(0,6)$
$\sigma _{a}$	Standard deviation of $\log (\alpha _{\text {H}_{k}})$	$\log (\text {h}^{-1})$	$\text {Half-Cauchy}(0,1)$
$\sigma _{b}$	Standard deviation of $\log (\alpha _{\text {T}_{k}})$	$\log (\text {h}^{-1})$	$\text {Half-Cauchy}(0,1)$
$\sigma _{m}$	Standard deviation of $\log (\mu _{k})$	$\log (\text {h}^{-1})$	$\text {Half-Cauchy}(0,1)$
$\phi _{k}$	Normalised deviation of $\log (\alpha _{\text {H}_{k}})$ from $\log (\alpha _{\text {H}})$	Dimensionless	${\mathcal {N}}(0,1)$
$\eta _{k}$	Normalised deviation of $\log (\alpha _{\text {T}_{k}})$ from $\log (\alpha _{\text {T}})$	Dimensionless	${\mathcal {N}}(0,1)$
$\psi _{k}$	Normalised deviation of $\log (\mu _{k})$ from $\log (\mu )$	Dimensionless	${\mathcal {N}}(0,1)$
$\pi$	Repellency parameter	Dimensionless	$(1 \!-\! \pi ) \sim \text {Lognormal}(0,5)$
$\kappa$	Killing/disarming parameter	Dimensionless	$\text {Lognormal}(0,5)$
$\rho$	Relative availability of trap	Dimensionless	$\text {Lognormal}(0,5)$

*k* is the index for a given night, as defined in Table 3

**Fig. 3:**
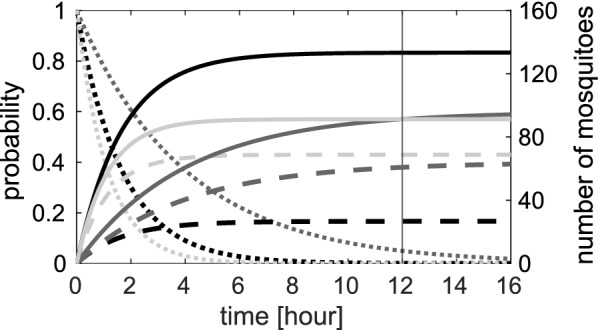
Made-up examples of host-seeking behaviour model without trap for three situations: high availability rate and low mortality rate (black), low availability and low mortality rate (dark grey), high availability and high mortality (light grey). The solid curves represent the probability $P_{\text {H}}(t)$ to get caught by HLC, the dashed curves represent the probability $P_{\text {M}}(t)$ to die and the dotted curves represent the probability $P_{\text {A}}(t)$ to be in the host-seeking state. All curves can also be interpreted as the corresponding expected cumulative count for 160 independent mosquitoes being released. The first situation (black) could be interpreted as host seeking on an unprotected host; the second (dark grey) as host seeking on a host protected by an intervention that repels mosquitoes; and the third situation (light grey) as host seeking on a host protected by an intervention that kills mosquitoes; the last two situations would be indistinguishable when only looking at the cumulative HLC counts after 12 h

For a given experimental night $k \in \{1, \ldots, 16\}$, a mosquito starts in host-seeking state (A) at the beginning of the experiment and can move to one of the three absorbing states, namely HLC (H), trap (T) or death/disarmed (M) at any time during the experiment. Note that death cannot be distinguished from being disarmed until the end of the night in the present experiment as either endpoints make the mosquito unresponsive to the HLC. Hence, both endpoints are combined into one definitive state. We assume that the probabilities $P_{\text {H}}(h), P_{\text {T}}(h), P_{\text {M}}(h)$ to move to the respective states within a short time *h* can be approximated linearly in time *h* with constant rates $\alpha _{\text {H}_{k}}$, $\alpha _{\text {T}_{k}}$ and $\mu _{k}$, respectively. These assumptions uniquely define a time-homogeneous, continuous-time Markov chain *X*(*t*) on the finite state space $\{\text {A}, \text {H}, \text {T}, \text {M}\}$ with probabilities $P_{\text {A}}(t)$ to stay in A, $P_{\text {H}}(t)$ to move to H, $P_{\text {T}}(t)$ to move to T and $P_{\text {M}}(t)$ to move to M within any time *t*, as illustrated in Fig. 2b. When plugging in the maximal host seeking duration per day for *t*, these probabilities correspond to the proportions in the deterministic model [25]. Alternatively, this stochastic process can be characterised as leaving state A with a negative exponentially distributed waiting time with mean $(\alpha _{\text {H}_{k}} + \alpha _{\text {T}_{k}} + \mu _{k})^{-1}$, and then picking one of the states H, T or M with probability $\tfrac{\alpha _{\text {H}_{k}}}{\alpha _{\text {H}_{k}} + \alpha _{\text {T}_{k}} + \mu _{k}}, \tfrac{\alpha _{\text {T}_{k}}}{\alpha _{\text {H}_{k}} + \alpha _{\text {T}_{k}} + \mu _{k}}$ or $\tfrac{\mu _{k}}{\alpha _{\text {H}_{k}} + \alpha _{\text {T}_{k}} + \mu _{k}}$, respectively. Elaboration of the Markov model including the equations for the probabilities $P_{\text {A}}(t), P_{\text {H}}(t), P_{\text {T}}(t)$ and $P_{\text {M}}(t)$ are provided in Additional file 1: Appendix A. For a situation without trap, the probability $P_{\text {H}}(t)$ of a mosquito to get caught by HLC is essentially determined by the human availability rate $\alpha _{\text {H}_{k}}$ controlling the speed of increase and by the mosquito mortality/disarming rate $\mu _{k}$ controlling how quickly it plateaus, as illustrated in Fig. 3.

Due to the time-homogeneity of the model, the probability that a given transition occurs between two time points only depends on the duration between the two time points, and not on their external clock time. Consequently, the probability to stay in state A until at least time $t_1$ and to then move to state H until at most time $t_2 \ge t_1$ is given by

1 $$\begin{aligned} P[X(t) = \text {A} \text { for } 0 \le t \le t_1, X(t_2) = \text {H}] = P_{\text {A}}(t_1) P_{\text {H}}(t_2 - t_1), \end{aligned}$$

and analogously for states A, T or M instead of H. Hence, we also have

2 $$\begin{aligned} P_{\text {A}}(l t) = P_{\text {A}}(t)^{l} \text { for any number l > 0 }. \end{aligned}$$

#### Multinomial likelihood for semi-field experiments over a single night

We denote the experimental data of a given night *k* by $D_{k} = (x_{\text {H1}}, x_{\text {H2}}, x_{\text {H3}}, x_{\text {H4}}, x_{\text {T}}, x_{\text {L}})$ (see Table 1 for notation). Our probability model assumes that all mosquitoes released during a single night in the same screen house behave independently and that all follow the same Markov model. As a consequence, $D_{k}$ follows a multinomial distribution, and we denote the corresponding probabilities with $p_{\text {H1}}$, $p_{\text {H2}}$, $p_{\text {H3}}$, $p_{\text {H4}}$, $p_{\text {T}}$ and $p_{\text {L}}$. These probabilities are given in terms of the probabilities of the Markov model, with time measured in hours, by use of Eqs. (1) and (2) as well as the requirement that they must sum up to 1:

3 $$\begin{aligned} \begin{aligned} p_{\text {H1}}&= P_{\text {H}}(1) \\ p_{\text {H2}}&= P_{\text {A}}(1) P_{\text {H}}(1) \\ p_{\text {H3}}&= P_{\text {A}}(1)^2 P_{\text {H}}(1) \\ p_{\text {H4}}&= P_{\text {A}}(1)^3 P_{\text {H}}(9) \\ p_{\text {T}}&= P_{\text {T}}(12) \\ p_{\text {L}}&= 1 - P_{\text {H}}(1) (1 + P_{\text {A}}(1) + P_{\text {A}}(1)^2) - P_{\text {A}}(1)^3 P_{\text {H}}(9) - P_{\text {T}}(12). \end{aligned} \end{aligned}$$

**Fig. 4:**
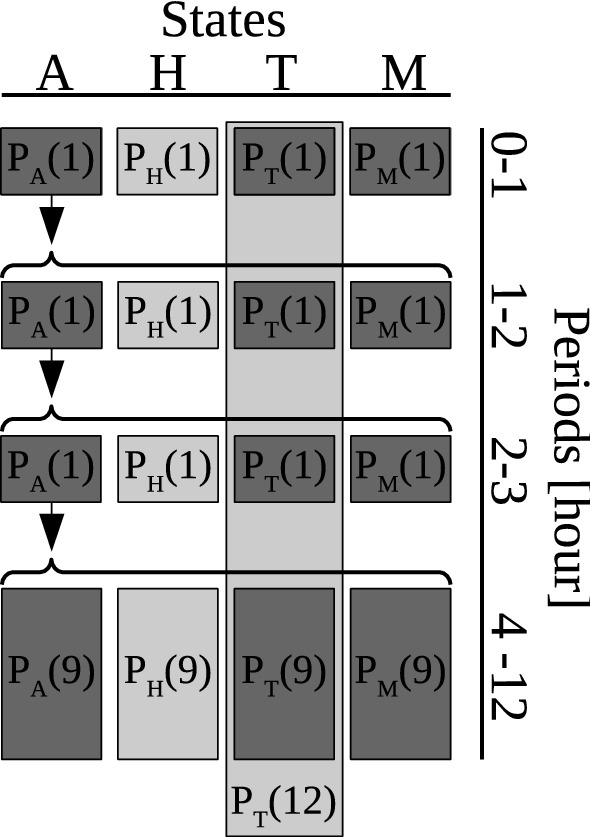
Schematic of multinomial model for semi-field experiments over a single night. Modelled outcomes and their probabilities are shown in a table, with columns representing the states host seeking (A), HLC (H), trap catch (T) and death (M), respectively, and rows representing the HLC periods during hours 0–1, 1–2, 2–3 and 3–12. Boxes in light grey and dark grey represent observed and unobserved outcomes, respectively. Note that only the cumulative trap catches over the whole night (hours 0–12) are observed. For notation of probabilities see Fig. 2b or Table 3

The link between the probabilities with lowercase *p* (multinomial model) and uppercase *P* (Markov model) is shown in Fig. 4. Note that the fourth HLC period was actually from hour 11.5 to 12, but is used as a proxy for HLC measured from hour 3 to 12.

Hence, the log-likelihood function for the semi-field model of a single night in terms of the rates $\alpha _{\text {H}_{k}}, \alpha _{\text {T}_{k}}, \mu _{k}$, given data $D_{k}$, is

4 $$\begin{aligned} l_{1}(\alpha _{\text {H}_{k}}, \alpha _{\text {T}_{k}}, \mu _{k} | D_{k}) & = c + x_{\text {H1}} \log (p_{\text {H1}}) + x_{\text {H2}} \log (p_{\text {H2}}) + x_{\text {H3}} \log ( p_{\text {H3}}) + x_{\text {H4}} \log (p_{\text {H4}}) \nonumber \\&\quad + x_{\text {T}} \log (p_{\text {T}}) + (n - x_{\text {H1}} - x_{\text {H2}} - x_{\text {H3}} - x_{\text {H4}} - x_{\text {T}}) \log (p_{\text {L}}), \end{aligned}$$

where *c* represents some constant and the probabilities are functions of the rates* via* Eq. (3) and the Markov model.

The likelihood function (Eq. 4) allows us to infer the shape of the expected HLC count curve (solid curves in Fig. 3) and hence to estimate the underlying parameters, including mortality or disarming, from time-stratified HLC data, despite a complete lack of direct experimental measurements. With only cumulative HLC counts over the night, we could not infer the availability and mortality/disarming rate, as illustrated in Fig. 3 with two different sets of parameters leading to the same expectation of cumulative HLC counts after 12 h.

#### Intervention parameterisation

The model described in Eq. (4) can be fitted to control and intervention experiments of a given night *k* separately, yielding control parameters $(\alpha _{\text {H}_{k}}[\text {C}], \alpha _{\text {T}_{k}}[\text {C}], \mu _{k}[\text {C}])$ and treatment parameters $(\alpha _{\text {H}_{k}}[\text {I}], \alpha _{\text {T}_{k}}[\text {I}], \mu _{k}[\text {I}])$. Even if the absolute values of these control and intervention parameters are unrealistic for field situations, a good metric for the difference between control and intervention parameters might be reasonably consistent between semi-field and field situations. Here, we introduce a set of parameters capturing the difference between the control and intervention parameters in order to parameterise the intervention and, ultimately, predict the effect in a field situations.

##### Repellency

To estimate repellency of a given intervention we take the relative reduction of the human availability rate:

5 $$\begin{aligned} \pi = 1 - \tfrac{\alpha _{\text {H}_{k}}[\text {I}]}{\alpha _{\text {H}_{k}}[\text {C}]} \in (-\infty , 1]. \end{aligned}$$

The reduction in availability can be due to diversion, masking human odours, confusing or some combination of these. Repellency is characterised by $\pi \in (0,1]$, while a tool with $\pi < 0$ would increase the attractiveness of the human host.

##### Killing and/or disarming effect during host-seeking state

To estimate the killing and/or disarming effect during the host-seeking state (referred to as the killing/disarming effect in the following text) of the given intervention, we take the difference of the mosquito mortality/disarming rates divided by the human availability in the control:

6 $$\begin{aligned} \kappa = \tfrac{\mu _{k}[\text {I}] - \mu _{k}[\text {C}]}{\alpha _{\text {H}_{k}}[\text {C}]} \ge 0. \end{aligned}$$

Negative values for $\kappa$ would indicate an increase in mosquito survival and are excluded due to requirements of the malaria simulation platform OpenMalaria [31], nevertheless they were allowed in the fitting procedure.

Note that an intervention with either $\pi < 0$ or $\kappa < 0$ may still reduce host-biting if it entails at the same time a strong killing/disarming effect or a strong repelling effect, respectively. Even an intervention with no direct mortality effect, i.e. $\kappa = 0$, but with a repelling effect, i.e. $\pi > 0$, will lower the mean mosquito life span, since in each feeding cycle the mosquitoes will spend more time in the host-seeking state where the mortality rate is usually higher than the average mortality rate over the other states.

##### Relative trap availability

To estimate the availability of a trap we take the ratio of the trap availability rate over the human availability in the control:

7 $$\begin{aligned} \rho = \tfrac{\alpha _{\text {T}_{k}}[\text {I}]}{\alpha _{\text {H}_{k}}[\text {C}]} \in [0, \infty ). \end{aligned}$$

If as many mosquitoes would be caught by a trap as would encounter a human, then this trap would be characterised by $\rho = 1$. We ignore potential interaction effects of the traps with the human availability rate or the mosquito mortality/disarming rate. An intervention consisting of a trap with $\rho > 0$ and otherwise no repelling or killing/disarming component, i.e. $\pi = \kappa =0$, reduces host biting since the trap availability rate acts as an additional mortality rate.

We can now give the joint log-likelihood for intervention data $D_{k}[\text {I}]$ and control data $D_{k}[\text {C}]$ for any intervention in terms of the intervention parameters $\pi , \kappa$ and $\rho$:

8 $$\begin{aligned}&l_{2}(\pi , \kappa , \rho , \alpha _{\text {H}_{k}}, \alpha _{\text {T}_{k}}, \mu _{k} | D_{k}[\text {I}], D_{k}[\text {C}]) \nonumber \\ & =l_{1}(\alpha _{\text {H}_{k}}, \alpha _{\text {T}_{k}}, \mu _{k} | D_{k}[\text {C}]) \\& + l_{1}((1 - \pi ) \alpha _{\text {H}_{k}}, \rho \alpha _{\text {H}_{k}}, \mu _{k} + \kappa \alpha _{\text {H}_{k}}| D_{k}[\text {I}]), \end{aligned}$$

where the first term on the right-hand side represents the control and the second term represents the intervention arm of the experiment. The parameters $\alpha _{\text {H}_{k}}$ and $\mu _{k}$ represent the control rates, but we omit the usual $[\text {C}]$ for notational convenience and since replicating the model over multiple nights with night-specific rates but constant intervention parameters will make $\alpha _{\text {H}_{k}}$ and $\mu _{k}$ also depend on the intervention experiment. It is necessary to include $\alpha _{\text {T}_k}[\text {C}]$ in the analysis of the control arm since unbaited control traps occasionally catch mosquitoes.

There are other possible metrics for the difference between control and intervention parameters; we chose metrics that are consistent with requirement of the malaria simulation platform OpenMalaria [31].

#### Hierarchical Bayesian statistical model for variation of experimental conditions over multiple semi-field nights

Semi-field experiments are typically replicated over multiple nights for statistical power and to account for night-specific variation in HLC response. We denote the control data over 16 nights by $D[\text {C}] = (D_{1}[\text {C}],\ldots,D_{16}[\text {C}])$ and the intervention data over the same 16 nights by $D[\text {I}] = (D_{1}[\text {I}],\ldots,D_{16}[\text {I}])$. We use a hierarchical Bayesian model to allow the rates to vary between nights on a scale inferred from the data itself. This is comparable to partial pooling with frequentist methods. For every night $k \in \{1, \ldots, 16 \}$ , we take an independent human availability rate $\alpha _{\text {H}_{k}}$, an independent trap availability rate $\alpha _{\text {T}_{k}}$ and an independent mortality/disarming rate $\mu _{k}$ with distributions

9 $$\begin{aligned} \alpha _{\text {H}_{k}}&\sim \text {Lognormal}(a, \sigma _{a}^{2}) \nonumber \\ \alpha _{\text {T}_{k}}&\sim \text {Lognormal}(b, \sigma _{b}^{2}) \nonumber \\ \mu _{k}&\sim \text {Lognormal}(m, \sigma _{m}^{2}), \end{aligned}$$

where *a*, *b* and *m* are unrestricted hyperparameters and $\sigma _{a}, \sigma _{b}$ as well as $\sigma _{m}$ are positive hyperparameters. For a single experimental arm (regardless of whether control or intervention) with data $D = (D_{1},\ldots,D_{16})$, we obtain by Bayes’ theorem the joint log-probability density function (log-posterior) of all parameters:

10 $$\begin{aligned}&\text {pr}[a, b, m, \sigma _{a}, \sigma _{b}, \sigma _{m}, \alpha _{\text {H}_{1}}, \ldots, \alpha _{\text {H}_{16}}, \alpha _{\text {T}_{1}}, \ldots, \alpha _{\text {T}_{16}}, \mu _{1}, \ldots, \mu _{16} | D] \nonumber \\&\quad = \sum _{k=1}^{16} \big \{ l_{1}(\alpha _{\text {H}_{k}}, \alpha _{\text {T}_{k}}, \mu _{k} | D_{k}) + \text {pr}[\alpha _{\text {H}_{k}} | a, \sigma _{a}] + \text {pr}[\alpha _{\text {T}_{k}} | b, \sigma _{b}] + \text {pr}[\mu _{k} | m, \sigma _{m}] \big \} \nonumber \\&\qquad + \text {pr}[a,b,m, \sigma _{a}, \sigma _{b}, \sigma _{m}] - c, \end{aligned}$$

where *c* is some normalisation term and $l_{1}$ is the likelihood function defined in Eq. (4). The hierarchical priors of the night-specific availability and mortality/disarming rates are given by Eq. (9) in terms of the hyperparameters. The log-probabilities on the last line are hyperpriors. The posterior distribution of the hyperparameters $a,b,m,\sigma _{a}, \sigma _{b}$ and $\sigma _{m}$ contain all information on the mean rates, i.e. the rates $\alpha _{\text {H}}, \alpha _{\text {T}}$ and $\mu$ of any further unknown night,* via *the expectation for the Lognormal distributions (Eq. 9):

11 $$\begin{aligned} \alpha _{\text {H}} &= \mathop {{}{\mathbb {E}}}[\alpha _{\text {H}_{k}}]= \exp (a + \sigma _{a}^2 /2) \nonumber \\ \alpha _{\text {T}} & = \mathop {{}{\mathbb {E}}}[\alpha _{\text {T}_{k}}]= \exp (b + \sigma _{b}^2 /2) \nonumber \\ \mu &= \mathop {{}{\mathbb {E}}}[\mu _{k}]= \exp (m + \sigma _{m}^2 /2). \end{aligned}$$

This is all we want to know for a specific arm of a semi-field experiment; the night-specific rates $\alpha _{\text {H}_{1}}, \ldots, \alpha _{\text {H}_{16}}$, $\alpha _{\text {T}_{1}}, \ldots, \alpha _{\text {T}_{16}}$ and $\mu _{1}, \ldots, \mu _{16}$ are nuisance parameters.

To parameterise an intervention from control data $D[\text {C}] = (D_{1}[\text {C}],\ldots,D_{16}[\text {C}])$ and intervention data $D[\text {I}] = (D_{1}[\text {I}],\ldots,D_{16}[\text {I}])$ over 16 nights, we use the same hierarchical model for the control rates. The hierarchical model agrees with the intervention parameterisation in the sense that Eqs. (5)–(7) also hold for the mean rates $\alpha _{\text {H}}, \alpha _{\text {T}}$ and $\mu$. In particular, we have $\mathop {{}{\mathbb {E}}}[\mu _k[\text {I}]] = \mu [\text {C}] + \kappa \alpha _{\text {H}}[\text {C}].$ Bayes’ Theorem gives the joint log-probability density function (log-posterior) of all parameters:

12 $$\begin{aligned}&\text {pr}[\pi , \kappa , \rho , a, b, m, \sigma _{a}, \sigma _{b}, \sigma _{m}, \alpha _{\text {H}_{1}}, \ldots, \alpha _{\text {H}_{16}}, \alpha _{\text {T}_{1}}, \ldots, \alpha _{\text {T}_{16}}, \mu _{1}, \ldots, \mu _{16} | D[\text {C}], D[\text {I}]] \nonumber \\&\quad = \sum _{k=1}^{16} \big \{l_{2}(\pi , \kappa , \rho , \alpha _{\text {H}_{k}}, \alpha _{\text {T}_{k}}, \mu _{k} | D_{k}[\text {I}], D_{k}[\text {C}]) \big \} \nonumber \\&\qquad + \sum _{k=1}^{16} \big \{ \text {pr}[\alpha _{\text {H}_{k}} | a, \sigma _{a}] + \text {pr}[\alpha _{\text {T}_{k}} | b, \sigma _{b}] + \text {pr}[\mu _{k} | m, \sigma _{m}] \big \} \nonumber \\&\qquad + \text {pr}[\pi , \kappa , \rho ,a,m, \sigma _{a}, \sigma _{m}] -c, \end{aligned}$$

where *c* is some normalisation term and $l_{2}$ is the likelihood function defined in Eq. (8). The hierarchical priors of the night-specific availability and mortality/disarming rates are given by Eq. (9) in terms of the hyperparameters. The log-probabilities on the last line are priors and hyperpriors. This approach matches control and intervention data by night, as we only allow the rates to vary by night, and not by experiment. Matching is suggested by the experimental design and justified since the volunteer and screen house are randomised.

#### Bayesian parameter inference for semi-field model

We use a Markov chain Monte Carlo (MCMC) method to generate a sample of the posterior distributions specified in Eqs. (10) and (12). Specifically, we use the Hamiltonian Monte Carlo method implemented in Stan [33, 34], which is advantageous for models with many parameters. Computations are performed with Rstan [35], the Stan integration in R [36]. To avoid parameter correlation originating from the hierarchical model structure (see [37] for consequent sampling problems), we implement non-central equivalents of the models in Eqs. (10) and (12) in Stan, as detailed in Additional file 1: Appendix B. The Stan code corresponding to Eq. 10 for a single experimental arm is provided in Additional file 2: S1 and Additional file 3: S2. The Stan code corresponding to Eq. 12 for both the intervention and control arm is provided in Additional file 4: S3, Additional file 5: S4 and Additional file 6: S5. We use the priors displayed in Table 5. We checked that posteriors of the parameters of interest do not change when varying the parameters of the priors in same range; in particular, much larger scale parameters were tested. We run four Markov chains, each with 6000 iterations, including 3000 for the burn-in. The diagnostics provided by Stan were used to confirm convergence of the chains; in particular, the $\hat{{R}}$-statistic was checked to be reasonably close to 1. We checked trace plots of the Markov chains to check mixing; and we plotted pairwise scatter plots of the posterior sample to check for non-correlation and to exclude potential identifiability problems (see Additional file 1: Appendix C.5 and D.5). One experimental night of the push–pull experiments, in which the number of mosquitoes recaptured was higher than the number of mosquitoes released, was discarded for the model fit.

For an unmatched analysis we fit Eq. (10) separately to each experimental arm (results shown in Additional file 1: Appendix C). We parameterise the repellent by fitting Eq. (12) to the repellent data while omitting all terms involving the trap, i.e. by setting $\rho , b, \sigma _b, \alpha _{\text {T}_1}, \ldots, \alpha _{\text {T}_16}$ all equal 0. The trap is parameterised by fitting Eq. (12) to the trap data while fixing $\pi = \kappa = 0$. Finally, we parameterise the push–pull system by fitting Eq. (12) to the push–pull data while setting $\rho$ to the value estimated from the trap data. Note that for the push–pull system the numbers of trap catches were only measured over 3 h instead of 12 h as for the trap experiments.

### Model for delayed mortality

To parameterise an intervention with potential killing effect, one also needs to quantify the postprandial killing effect, i.e. the killing effect on mosquitoes after they have fed on a human host protected by the tool. It is important to distinguish between mortality before and after biting because of their differential impact on transmission and because of different exposure intensities to repellent products during host seeking and while biting. We use a hierarchical binomial model for the delayed mortality data from Bagamoyo to infer the postprandial killing effect of the spatial repellent. All notations and all parameters for this model are shown in Table 6.

**Table 6:** Data variables, dependent parameters and independent parameters of model for delayed mortality and delayed killing effect

Symbol	Description	Unit	Prior
*k*	Index for night, $k \in \{1,\ldots ,16\}$	Integer	
$y_{k}$	Number of mosquitoes caught by HLC over all HLC periods in night *k*	Integer	
$z_{k}$	Number of mosquitoes caught by HLC over all HLC periods in night *k* and dead 12 h after the end of the semi-field experiment	Integer	
*y*	Collection of $y_{k}$ over all nights	Integer array	
*z*	Collection of $z_{k}$ over all nights	Integer array	
$p_{k}$	Probability of a mosquito that is caught by HLC in night *k* to be dead 12 h after the end of the semi-field experiment	Probability	
$q_{k}$	Probability of a mosquito that is caught by HLC in night *k* to be alive 12 h after the end of the semi-field experiment	Probability	
*p*	Mean of $p_{k}[\text {C}]$	Probability	
*r*	Mean of $\text {logit}(p_{k}[\text {C}])$	Real	$\text {Logistic}(0,1)$
$\sigma _{r}$	Standard deviation of $\text {logit}(p_{k}[\text {C}])$	Positive real	$\text {Half-Cauchy}(0,1)$
$\omega _{k}$	Normalised deviation of $\text {logit}(p_{k}[\text {C}])$ from *r*	$\text{Real}$	${\mathcal {N}}(0,1)$
$\xi$	Postprandial killing effect	Real $\in (-\infty , 1]$	$\text {Uniform}(0, 1)$

Data variables and parameters may be equipped with $[\text {C}]$ or $[\text {I}]$ to denote a control or intervention arm, respectively

For a given night *k*, let $y_{k}$ denote the number of mosquitoes caught by HLC over all HLC periods and let $z_{k}$ denote the number of mosquitoes among $y_{k}$ that are dead 12 h after the end of the semi-field experiment. We model

13 $$\begin{aligned} z_{k} \sim \text {Bin}(y_{k}, p_{k}), \end{aligned}$$

where Bin stands for the binomial distribution and $p_{k}$ denotes the probability for a mosquito that is caught by HLC to be dead 12 h after the end of the semi-field experiment in night *k*. We use $q_{k}$ to denote the complementary survival probability, i.e. the probability for a mosquito that is caught in HLC to be alive 12 h after the end of the semi-field experiment in night *k*. The corresponding likelihood function is

14 $$\begin{aligned} l(p_{k} | y_{k}, z_{k}) = { y_{k} \atopwithdelims ()z_{k} } p_{k}^{z_{k}} (1-p_{k})^{y_{k} - z_{k}}. \end{aligned}$$

We then define the postprandial killing effect $\xi$ to be the real number in [0, 1] such that

15 $$\begin{aligned} q[\text {I}]_{k} = (1 - \xi ) q[\text {C}]_{k}, \end{aligned}$$

where $q[\text {I}]_{k} = 1 - p[\text {I}]_{k}$ denotes the survival probability in the intervention experiment and $q[\text {C}]_{k} = 1 - p[\text {C}]_{k}$ denotes the survival probability in the control experiment. Equivalently, we have

16 $$\begin{aligned} p[\text {I}]_{k} = p[\text {C}]_{k} + \xi ( 1 - p[\text {C}]_{k}). \end{aligned}$$

To allow these probabilities to vary among multiple nights with a scale inferred from the data itself, we set

17 $$\begin{aligned} p[\text {C}]_{k} = \text {logit}^{-1}( r + \omega _{k} \sigma _{r}) \quad \text {with} \quad \omega _{k} \sim {\mathcal {N}}(0,1), \end{aligned}$$

where *r* is a unrestricted real number, $\omega _{k}$ is a random variable and $\sigma _{r}$ is a positive real number. The expectation of the death probabilities in a control and in an intervention experiment are then

18 $$\begin{aligned} p[\text {C}] = {\mathbb {E}}(p[\text {C}]_{k}) \quad \text {and} \quad p[\text {I}] = p[\text {C}] + \xi (1 - p[\text {C}]), \end{aligned}$$

respectively, which correspond to $P_{B \mu }$ from [25] for control and intervention human-host type.

By Bayes’ theorem we infer the log-probability density function (log-posterior) of the parameters from the data as

19 $$\begin{aligned}&\text {pr}[\xi , r, \sigma _{r}, \omega _{1}, \ldots, \omega _{16} |y[\text {C}], z[\text {C}], y[\text {I}], z[\text {I}] ] \nonumber \\&\quad = \sum _{k=1}^{16} \Big \{ l\big ( p[\text {C}]_{k} | y[\text {C}]_{k},z[\text {C}]_{k} \big ) \text {pr}[\omega _{k}] + l\big ( p[\text {I}]_{k} | y[\text {I}]_{k},z[\text {I}]_{k} \big ) \text {pr}[\omega _{k}] \Big \} \nonumber \\&\qquad + \text {pr}[\xi , r, \sigma _{r}] -c, \end{aligned}$$

where the likelihood function *l* is defined in Eq. (14) and $p[\text {C}]_{k}$ as well as $p[\text {I}]_{k}$ are given by Eqs. (16) and (17). The log-probabilities for $\omega _{k}$ are given by their standard-normal distribution, while for $\xi , r$ and $\sigma _{r}$ , priors are chosen according to Table 6. The parameter *r* is given a $\text {logistic}(0,1)$ prior so that $p_{k}[\text {C}]$ has a nearly uniform prior, while slightly fatter boundaries are due to the hierarchical term $\omega _{k} \sigma _{r}$. We fit this model with the same methodology as described above for the SFS model. The corresponding stan code is provided in Additional file 7: S6 and Additional file 8: S7. Negative values for $\xi$ would indicate a reduction of the death probability due to the intervention and are excluded for fitting reasons. We use the same parameterisation for the push–pull intervention because these experiments were only conducted for the spatial repellent.

### Effect on vectorial capacity

#### Relative reduction of vectorial capacity

Vectorial capacity is a measure of the ability of the vector population to transmit malaria that is independent of the infectiousness of humans. It was originally defined as ‘the average number of inoculations with a specified parasite, originating from one case of malaria in unit time, that a vector population would distribute to man if all the vector females biting the case became infected’ in [38]. We compute the vectorial capacity at steady state of the deterministic ‘malaria in mosquito’ model [see [25], Eq. (20)]. To quantify the effect of the repellent, trap and push–pull system deployed at a given coverage level, we use the relative reduction of vectorial capacity in this situation* versus* the baseline vectorial capacity. The relative reduction of vectorial capacity is independent of the larval carrying capacity of the environment since the vectorial capacity is proportional to the mosquito emergence rate.

#### Baseline for entomological setting

For the baseline we use the parameters specific to *An. arabiensis* as published in the supplement (see Table 8) to [39]. We display all baseline parameters in Table 7, denoted with a subscript $\text {b}$ and converted to time measured in hours.

**Table 7:** Baseline parameters for deterministic ‘malaria in mosquito’ model for species *Anopheles arabiensis*

Symbol	Description	Value	Unit
$N_{}$	Total number of human hosts	100,000	Animals
$\alpha _{\text {H}_{\text {b}}}$	Availability rate of human hosts covered only by baseline interventions to mosquitoes of species *An. arabiensis*	3.75E−07	$\text {h}^{-1}$
*H*	Average number of humans per household	5	Animals
$\mu _{vA}$	Per-capita mosquito death rate while searching for a blood meal for the mosquito species *An. arabiensis*	0.01	$\text {h}^{-1}$
$\theta _{d}$	Maximum length of time that a mosquito searches for a host in 1 day if it is unsuccessful	8	h
$P_{B_{\text {b}}}$	Probability that a mosquito bites after encountering a host covered only by baseline interventions	0.95	Dimensionless
$P_{C_{\text {b}}}$	Probability that a mosquito finds a resting place after biting a host covered only by baseline interventions	0.95	Dimensionless
$P_{D_{\text {b}}}$	Probability that a mosquito survives the resting phase after biting a host covered only by baseline interventions	0.99	Dimensionless
$P_{E_{\text {b}}}$	Probability that a mosquito lays eggs and returns to host-seeking after biting a host covered only by baseline interventions	0.88	Dimensionless
$\tau$	Time required for a mosquito that has encountered a host to return to host seeking (provided that the mosquito survives to search again)	72	h
$\theta _{s}$	Duration of the extrinsic incubation period. This is the time required for sporozoites to develop in the mosquito	264	h

All values are taken from Table 8 in the supplement to [39], with rates being converted from $\text {day}^{-1}$ to $\text {h}^{-1}$

These parameters assume the role of the control parameters from the semi-field experiments, but in contrast to the semi-field parameters they represent a realistic field situation.

#### Implementation of parameterisation of interventions

Adding to the baseline situation one of the vector control intervention means specifying a host type for protected humans with availability rate $(1 - \pi ) \alpha _{\text {H}_{\text {b}}}$ as well as proportion $(1 - \xi )P_{C_{\text {b}}}$ of mosquitoes successfully biting, a shadow host type with availability rate $\kappa \alpha _{\text {H}_{\text {b}}}$ and a dummy host type with availability rate $\rho \alpha _{\text {H}_{\text {b}}}$ and mosquito death proportion after encountering $P_{B_{\text {T}} \mu } = 1$ (if traps are deployed). Killing or disarming are modelled by either killing all mosquitoes that encountered a shadow host or by keeping them in the host-seeking state *A*, respectively. To simulate repellent, traps and push–pull, the corresponding parameterisation is sampled from the posterior of the corresponding intervention parameterisation. The number of human hosts protected by a certain intervention, as well as the number of corresponding shadow hosts, is given by the total number of human hosts (*N*) multiplied with the coverage level. The number of traps is obtained by multiplying the coverage level with the total number of human hosts, divided by the average household size (*H*).

## Results

We present an analysis including mortality and disarming of the semi-field experimental data published in [16], a parameterisation for the spatial repellent, trap and push–pull intervention learned from this data, and our prediction of the impact of these tools on vectorial capacity.

### Data and model prediction for semi-field experiments

**Fig. 5:**
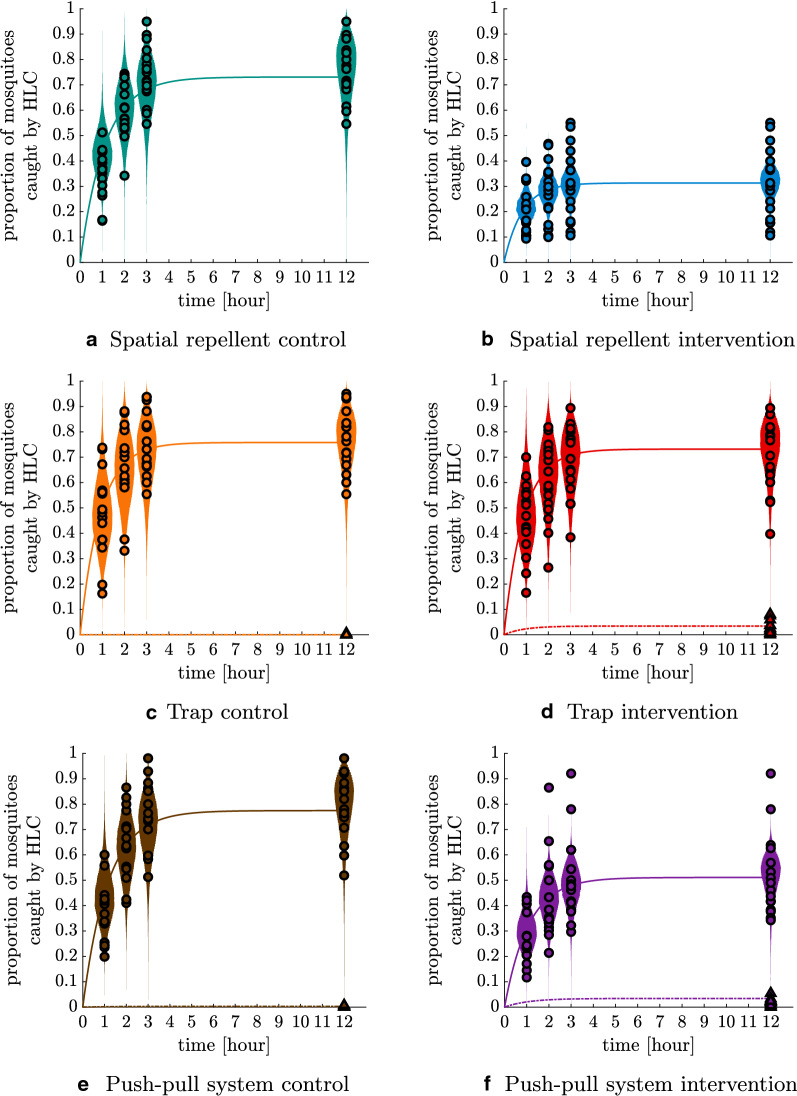
Data and model prediction by the matched SFS model for all arms of the semi-field experiments from Mbita. Circles represent the proportion of mosquitoes caught by HLC in a specific night of the given experiment from the beginning of the night until the hour indicated on the horizontal axes. Note that the circles at hour 12 are the proportions of the HLC until hour 3 together with the HLC from hour 11.5 until hour 12, as this is used as a proxy for the HLC during the whole night. Triangles represent the proportion of mosquitoes caught by the trap in a specific night of the given experiment during the whole night. All proportions are with respect to the the number of mosquitoes released in the corresponding night. Solid curves represent the expectation of cumulative HLC counts over continuous time and dashed curves denote the expectation of cumulative trap counts over continuous time, as estimated with a model matching control and intervention experiments per night. The coloured areas represent histograms of simulated data by the fitted model. Per experiment, an average of 2428 mosquitoes in total was released (2370–2474).** a**,** b** In total, 1778 (**a**) and 771 (**b**) mosquitoes were caught by HLC in these experiments.** c**–**f** In total, 1843 (**c**), 1715 (**d**), 1980 (**e**) and 1262 (**f**) mosquitoes were caught by HLC, respectively, in these experiments, and a total of 1, 16, 6 and 46 mosquitoes, respectively, were caught in the trap

Figure 5 shows the cumulative HLC counts and the trap catches for each night as proportions with respect to the number of mosquitoes released in each night as given in the Mbita data. Control and intervention experiments were run with 160 mosquitoes each per night, but only the mosquitoes which left the release cup were counted as released. The model took into account the specific HLC count pattern of each single night, while the night-specific data is not shown here. These data are underlaid with the prediction by the matched SFS model (see Eq. 12) fitted to the data: the mean probability (i.e. expected proportion) that a mosquito gets caught by HLC or by the trap plotted as a continuous curve over time, and smoothed histograms of simulated data as a posterior predictive check. The smoothed histograms were produced by the function distributionPlot in MATLAB [40]. In Fig. 5f, the three topmost data points all belong to the experimental night with the lowest number of mosquitoes released in both arms (126 mosquitoes* vs* a mean of 152 in the intervention arm and 140 mosquitoes* vs* a mean of 154 in the control). A possible explanation for these outliers could be that mosquitoes were more starved in that night compared to other nights, increasing the relative HLC response. Also, smaller release numbers are associated with higher variability relative to the mean. Night 10 of the push–pull experiments was discarded for the model fit since the number of mosquitoes recaptured was higher than the number of mosquitoes released.

Figure 5 highlights the concept of the semi-field model: The mortality/disarming rate can be inferred from the shape of the HLC count pattern over time. In particular, the higher the mortality and the disarming, the quicker the curve of expected proportion of HLC counts plateaus, indicating that no mosquito is left to respond. If only the cumulative HLC count per night was known, no information on mortality or disarming during host seeking could be inferred.

### Parameterisation of semi-field experiments

**Fig. 6:**
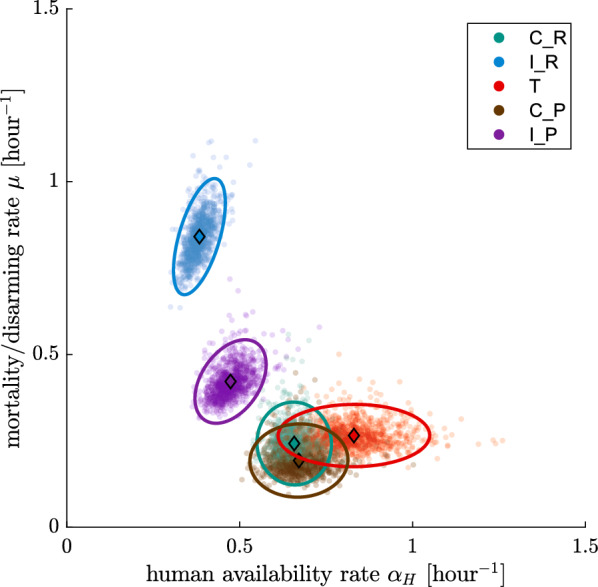
Characterisation of separate semi-field experiments from Mbita in terms of human availability rate ($\alpha _{\text {H}}$) and mosquito mortality/disarming rate before host encounter ($\mu$), as estimated with a model matching control and intervention experiments per night: spatial repellent control ($\text{C}_\text{R}$, green) and intervention ($\text{I}_\text{R}$, blue), trap control and intervention together (*T*, red), as well as push–pull control ($\text{C}_\text{P}$, brown) and intervention ($\text{I}_\text{P}$, purple). The parameterisation for trap control and intervention experiments are perfectly overlapping in terms of $\alpha _{\text {H}}$ and $\mu$ since they the trap is solely modelled by its relative availability, and not by a change of $\alpha _{\text {H}}$ or $\mu$. Each characterisation consists of a sample of 1000 points from the posterior (transparent points), the mean of the posterior (diamonds) and the 95% credible region with highest density after normal approximation of the posterior (ellipses)

Figure 6 shows the parameter inference for all semi-field experiments with respect to the human availability rate ($\alpha _{\text {H}}$) and the mosquito mortality/disarming rate before host encounter ($\mu$), by use of the semi-field model matching control and intervention (see Eq. 12). For the trap experiments, control and intervention parameterisation are identical with respect to human availability rate and mortality/disarming rate, so that only one posterior, denoted with ‘T’, is plotted. There are clearly three statistically significant clusters: repellent intervention ($\text {I}_{\text {R}}$), push–pull intervention ($\text {I}_{\text {P}}$) and the rest, consisting of the trap intervention and all control experiments. Even though not identical, the parameterisations of the control experiments all overlap substantially. The credible regions for the control experiments do not overlap completely, possibly because of the different weather conditions at the times of the year when the experiments were conducted (experiments were conducted in blocks) or because the control interventions (unbaited trap with working fan, untreated eave ribbon) had a minor effect. In Additional file 1: Appendix C.2, separate figures for each rate parameter inference are provided, by use of the same model as well as by use of the model without matching control and intervention, for comparison.

**Fig. 7:**
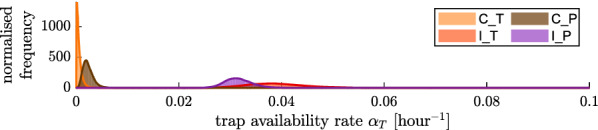
Trap availability rate $\alpha _{\text {T}}$ for all experiments involving traps from Mbita, as estimated with a model matching control and intervention experiments per night: trap control ($\text{C}_\text{T}$, orange) and intervention ($\text{I}_\text{T}$, red), as well as push–pull control ($\text{C}_\text{P}$, brown) and intervention ($\text{I}_\text{P}$, purple), presented as histograms of the posteriors (shaded areas) and probability density functions fitted to the posteriors with normal kernels (solid lines)

Figure 7 shows the trap availability rates for all control and intervention experiments involving unbaited and baited traps, respectively, as estimated with the model matching control and intervention (see Eq. 12). The scale of the horizontal axes is one order of magnitude smaller than that shown in Fig. 6. The availabilty rates of the baited traps are approximately 20-fold smaller than the human availability rates estimated from the respective control experiments. The availabilty rates of the unbaited traps are not exactly 0, but they are close to 0, as the unbaited traps occasionally caught mosquitoes.

### Intervention parameterisation

The intervention parameterisation for the spatial repellent (R), trap (T) and push–pull system (P) is presented in Table 8. All parameters except for $\xi$ are fitted to data from Mbita, with $\xi$ fitted to data from Bagamoyo as shown below.

**Table 8:** Intervention parameterisation: repelling effect ($\pi$), killing/disarming effect during host seeking state ($\kappa$), relative trap availability ($\rho$) and postprandial killing effect ($\xi$)

Parameter	Spatial repellent (R)	Trap (T)	Push–pull (P)
$\pi$	0.416 [0.35, 0.478]	–	0.294 [0.231, 0.354]
$\kappa$	0.911 [0.795, 1.035]	–	0.341 [0.288, 0.398]
$\rho$	–	0.047 [0.037,0.058]	0.047 [0.037, 0.058]
$\xi$	0.067 [0.013, 0.127]	–	0.067 [0.013, 0.127]

Each entry consists of the mean of the marginal posterior distribution (minimum mean squared error estimator) and the 95% credible interval of the form $[2.5 \text { percentile}, 97.5 \text { percentile}]$ of the marginal posterior distribution. All parameters except for $\xi$ are fitted to data from Mbita; $\xi$ is fitted to data from Bagamoyo. The estimate of $\xi$ obtained from the spatial repellent experiments is also taken to parameterise the push–pull system, as the push–pull experiments were not concluded in Bagamoyo

Each intervention is parameterised by a subset of four parameters:repelling effect ($\pi$), killing/disarming effect during host seeking state ($\kappa$), relative trap availability ($\rho$) and postprandial killing effect ($\xi$). Note that killing cannot be distinguished from disarming with the available data so that both effects are combined. The mean and the 95% credible interval of the form $[2.5 \text { percentile}, 97.5 \text { percentile}]$ of the marginal posterior distribution is given for each parameter. The definition of a credible interval is that the parameter value lies inside the interval with a probability of 95% given the observed data, but the given credible interval is generally not the shortest such interval (in contrast to highest density intervals). The push–pull parameterisation is designed as a trap intervention, with parameters estimated from the trap-only experiments together with a special spatial repellent intervention parameterised for push–pull and therefore shares the relative trap availability ($\rho$) with the trap intervention.

**Fig. 8:**
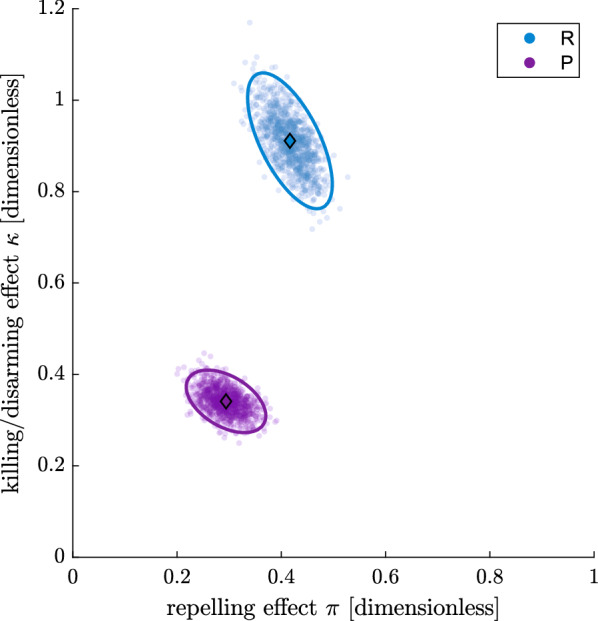
Characterisation of the spatial repellent (R) and push–pull (P) intervention in terms of repelling effect ($\pi$) and killing/disarming during the host-seeking state effect ($\kappa$), as estimated with a model matching control and intervention experiments per night from the data from Mbita. Each characterisation consists of a sample of 1000 points from the posterior (transparent points), the mean of the posterior (diamonds) and the 95% credible region with highest density after normal approximation of the posterior (ellipses)

Figure 8 shows the inference on the intervention parameterisation for the spatial repellent (R) and push–pull system (P) in the $\pi$–$\kappa$ plane. The scale for $\pi$ goes from ‘no change of human availability to mosquitoes’ (0) to ‘complete suppression of human availability to mosquitoes’ (1). The scale for $\kappa$ goes from ‘no increase of mosquito mortality or disarming before host encounter’ (0), to ‘increasing the mosquito mortality or disarming before host encounter by 1.2-fold the control human availability rate’ (1.2). The shapes of the credible regions (ellipses) clearly reveal a negative correlation between repelling effect ($\pi$) and killing/disarming effect ($\kappa$). In other words: within some small range, the model can barely distinguish between repellency ($\pi$) and mortality or disarming ($\kappa$). This is not surprising as increasing $\pi$ and increasing $\kappa$ both lower the probability to encounter a host. However, this does not constitute an identifiability problem since the credible regions are reasonably small.

**Fig. 9:**
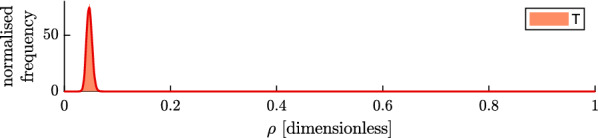
Relative trap availability ($\rho$) for trap intervention (T, red), as estimated with a model matching control and intervention experiments per night from the data from Mbita, presented as a histogram of the posterior (shaded area) and a probability density function fitted to the posterior with normal kernels (solid line)

Figure 9 shows the inference on the relative trap availability ($\rho$) for the trap experiments. A relative trap availability of $\rho =0$ would mean that mosquitoes are caught in traps with probability 0, and $\rho =1$ would mean that mosquitoes get caught in traps with the same probability as they encounter human hosts.

### Parameterisation for delayed mortality and postprandial killing effect

**Fig. 10:**
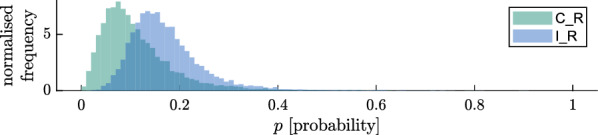
Delayed death probability (*p*) for repellent-control ($\text {C}_{\text {R}}$, green) and repellent-intervention ($\text {I}_{\text {P}}$, blue) experiments conducted in Bagamoyo, presented as histograms of the posteriors (shaded areas), as estimated with a model matching control and intervention experiments per night

**Fig. 11:**
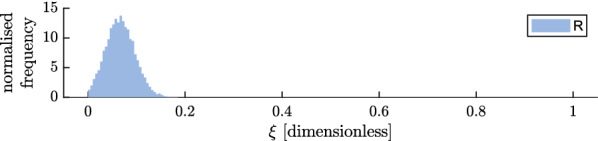
Postprandial killing effect ($\xi$) for the repellent intervention (R, blue) tested in Bagamoyo, presented as a histogram of the posterior (shaded area), as estimated with a model matching control and intervention experiments per night

Figure 10 shows the estimates of delayed mortality for the Bagamoyo data, and Fig. 11 shows the corresponding postprandial killing effect ($\xi$) of the spatial repellent, which is also summarised in Table 8. The scale for $\xi$ goes from ‘no increase of death probability after biting’ (0) to ‘probability 1 that mosquitoes die after biting’. Additional file 1: Appendix D.2 shows a corresponding figure by use of the model without matching control and intervention is provided for comparison.

### Prediction for relative reduction of vectorial capacity of *An. arabiensis*

**Fig. 12:**
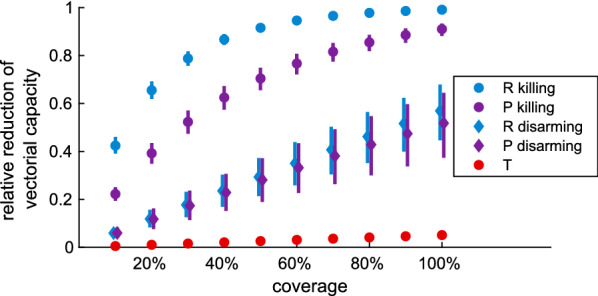
Prediction for relative reduction of vectorial capacity of *An. arabiensis* under deployment of spatial repellents (R, blue), traps (T, red) and push–pull systems (P, purple) at coverage levels ranging from 10 to 100%, under two distinct assumptions on the effect of the transfluthrin-treated eave ribbons: repelling and killing (circle markers), or repelling and disarming (diamond markers). Indoor biting is assumed to be equally affected by the interventions as outdoor biting. Markers denote means and vertical bars denote 95% credible intervals from 2.5 percentile to 97.5 percentile

In Fig. 12 we predict the relative reduction of vectorial capacity of *An. arabiensis* if spatial repellents (R), traps (T) or push–pull systems (P) are deployed at different coverage levels, based on the parameterisation obtained from the semi-field experiments. We present a scenario where $\kappa$ exclusively describes killing, corresponding to the assumption that all mosquitoes who stop responding to HLC in the semi-field experiments due to the intervention are dead, and a scenario where $\kappa$ exclusively describes disarming, corresponding to the assumption that all mosquitoes who stop responding to HLC in the semi-field experiments due to the intervention are disarmed. At 70% coverage with the spatial repellent, the relative reduction of the vectorial capacity of *An. arabiensis* falls with 95% probability between 96 and 97% (mean 97%) under the killing scenario, and between 31 and 50% (mean 41%) under the disarming scenario. The relative reduction by the same coverage with the push–pull system falls with 95% probability between 78 and 85% (mean 82%) under the killing scenario, and between 26 and 48% (mean 38%) under the disarming scenario. The relative reduction by the trap falls with 95% probability between 3 and 4% (mean 4%). In Additional file 1: Appendix E we provide an additional scenario assuming disarming mosquitoes for 3 days instead of 1 day, which shows no significant difference in terms of vectorial capacity. Note that the credible intervals of the effect on vectorial capacity only reflect the uncertainty of the intervention parameterisation and that no uncertainty comes from the mosquito life-cycle or dynamics of malaria in mosquitoes, since the ‘malaria in mosquito’ model is completely deterministic. Interventions are maintained at the indicated coverage level without decay, and the effect is estimated at steady state of the ‘malaria in mosquito’ population model [25].

### Further findings and inference diagnostics

Figures on intermediate parameters are presented in Additional file 1: Appendix C.1 for the semi-field model and Additional file 1: Appendix D.1 for the delayed mortality model. Graphics on the nightly variation are presented in Additional file 1: Appendices C.3 as well as C.4 for the semi-field model, and Additional file 1: Appendices D.3 and D.4 for the delayed mortality model. Correlation diagnostics of the Bayesian inference are presented in Additional file 1: Appendix C.5 for the semi-field model and Additional file 1: Appendix D.5 for the delayed mortality model.

## Discussion

### Impact on transmission of spatial repellent, trap and push–pull system tested in semi-field experiments

Recent semi-field experiments in Kenya and Tanzania measured the impact of a spatial repellent (transfluthrin-treated eave ribbons), an odour-baited trap (Suna trap baited with MB5 and carbon dioxide from molasses fermentation) and a push–pull system (combination of spatial repellent and trap) on human landing rates of *An. arabiensis* in the peridomestic area. The analysis of the Kenyan data showed that the spatial repellent had a strong impact, the push–pull system a more moderate impact and the trap no impact on personal protection, as measured in terms of human landing rates [16]. Complementing these findings in terms of community-level impact, our model estimates from the same data suggested that the spatial repellent, as well as the push–pull system, can reduce vectorial capacity of *An. arabiensis* substantially, assuming that the semi-field system represents the field well enough and that the SFS model is valid, as discussed in the model limitations below.

We choose relative reduction of vectorial capacity as a measure of transmission intensity since it allows us to estimate the effect of an intervention regardless of the local absolute mosquito abundance and is therefore suitable for comparison across settings with same vector and host–vector contact characteristics but different absolute biting numbers. Relative reduction of vectorial capacity accounts for the full decline of the ability of the vector population to transmit malaria to humans (both users and non-users of the intervention) and hence includes both personal and community protection. Correspondingly, we do not make statements on the impact on the absolute transmission intensity of the intervention at hand. Moreover, even though closely related to the basic reproduction number $R_0$, vectorial capacity cannot directly supply a criterion for whether the disease will die out or not.

#### Spatial repellent and push–pull system

This methodology allocates the effect of the spatial repellent to repellency on the one hand and mortality or disarming (preventing mosquitoes from host seeking) until the next night on the other. Disarming has a higher impact on transmission than repelling (though a lower impact than killing mosquitoes) and does not increase the risk for non-users. We found that the transfluthrin-treated eave ribbons reduced outdoor HLC to a large extent by killing or disarming mosquitoes until the next night, and hence can provide both user- and community-level protection. We underline that the model can only differentiate repellency from disarming or killing, but it cannot distinguish between disarming and killing, given the available semi-field data. Therefore, a scenario assuming that the transfluthrin-treated eave ribbons act through repelling and disarming (instead of killing) and a scenario assuming killing instead of disarming were considered, so that the corresponding estimates of the relative reduction of vectorial capacity of *An. arabiensis* can be interpreted as a lower and upper bound, respectively. Under the disarming assumption, the spatial repellent and the push–pull system are both predicted to substantially reduce the vectorial capacity of *An. arabiensis* at a realistic scale-up, and there is no significant difference between these two interventions over the whole range of coverage levels. Under the killing assumption, and assuming generalisability to the field, the spatial repellent can reduce the vectorial capacity of *An. arabiensis* drastically even at relatively low coverage, while the push–pull system achieves a similar reduction only at moderate to high coverage. Once the ratio between disarming and killing for a given intervention is known, the actual ratio can readily be incorporated into the vectorial capacity estimation. To investigate disarming* versus* killing, similar semi-field experiments in smaller compartments allowing all mosquitoes, including dead ones, to be recovered were conducted and are currently being analysed (personal communication, Mgeni M. Tambwe).

We found that although spatial repellents and the push–pull system exhibited similar levels of repellency, the spatial repellent had a much stronger killing/disarming effect. This explains the superiority of the spatial repellent over the push–pull system in reducing vectorial capacity under the killing assumption. However, under the disarming assumption, the push–pull system can compensate for its lower disarming effect with the killing effect of the trap and achieves the same levels of vectorial capacity reduction as the spatial repellent. One potential hypothesis for this effect is that push–pull has a lower killing/disarming effect because the trap lures the mosquitoes away from the human who is sitting close to the spatial repellent and therefore reduces mosquito exposure to the transfluthrin while, in comparison, the trap only catches a small proportion of the mosquitoes it lures away. This scenario would be in line with findings that the Suna trap provides a high level of attraction but a relatively low capture efficiency [41]. However, the higher killing/disarming effect of the spatial repellent compared to the push–pull system is also likely to be partially due to the spatial repellent experiments being conducted in a season with higher nightly temperatures than when the push–pull experiments were conducted [16], since evaporation of transfluthrin increases with temperature [14, 42] and higher concentrations are likely associated with a higher killing/disarming effect.

#### Trap

Our analysis showed that the trap has only a very minor effect on the vectorial capacity of *An. arabiensis*, although this may be only true for the mosquito species *An. arabiensis* and the setting tested in the experiments. Especially in view of the much higher costs associated with odour-baited traps compared to treated eave ribbons, these findings cannot justify the use of the trap in this setting. However, in contrast to the analysis in [16], we found the trap effect to be at least statistically significant in terms of both mosquito landing rates and vectorial capacity of *An. arabiensis*. The push–pull system under consideration was optimised in terms of its components (such as the repellent and trap used—see [16] for more details), but not in terms of the location, height and orientation of the trap. This, in addition to all experiments only being conducted on *An. arabiensis*, may explain why the trap was not as effective as it had been shown to be in previous studies [43].

#### Postprandial killing

We found the postprandial killing effect of the spatial repellent to be relatively small compared to the preprandial killing effect. The postprandial killing effect was only analysed for the spatial repellent, and the same value was used to estimate the reduction in vectorial capacity for the push–pull system, since delayed mortality experiments were not conducted for push–pull. The postprandial killing effect ($\xi$) was estimated from experiments conducted in Bagamoyo under different climatic and weather conditions. Hence, it may not be appropriate to couple parameter estimates from these different sites for the vectorial capacity predictions. Since we restricted the parameter of the postprandial killing effect to values indicating a positive killing effect ($\xi \in [0,1]$), we precluded possible negative values, and therefore the corresponding credible interval is biased to the right. Hence, the signal of the postprandial killing effect might actually not be statistically significant when relying on 95% credible intervals. Moreover, delayed mortality counts in the data might be too high, since in the experiments all HLC collection cups were placed directly beside the volunteer—i.e. close to the transfluthrin-treated eave ribbon—until the end of the third HLC period, and mosquitoes were therefore exposed to insecticides longer than in a field situation. If such data were collected in the future, we believe it would be important to bring mosquitoes outside of the effective zone of the insecticide immediately after completion of each HLC collection in order not to overestimate the delayed killing effect. Measuring delayed mortality 12 h after the end of the semi-field is arbitrary and might not be sufficient to capture the full delayed killing effect of the intervention. If the time to bite would differ significantly between treatment and control, measuring mortality after biting over a fixed time counted from the start of the experiment could overestimate mortality in the group with earlier biting times.

#### Scale-up

Overall, the results of this analysis suggest that transfluthrin-treated eave ribbons can be a promising tool against residual malaria transmission in the peridomestic area, providing both user and community protection, at least in areas where *An. arabiensis* is the dominant vector. Assuming generalisability of these findings to the field, it seems unnecessary to use a push–pull system in this setting as the rational of combining a spatial repellent with an outdoor trap is to kill repelled mosquitoes and therefore compensate for a potential adverse community-effect of the spatial repellent. These findings are drawn under the assumption that indoor biting is reduced at least as much as outdoor biting or that *An. arabiensis* is predominantly feeding outdoors, which may be the case in some areas [44].

However, an important drawback of testing spatial repellents in semi-field experiments is that mosquitoes cannot be pushed beyond the semi-field area. Hence, mosquitoes may be forced to stay in the semi-field area, whereas they would likely leave this area in a field situation. If they keep host seeking on the only available host, then the model estimates repellency correctly by the delayed HLC response, which is a considerable improvement over analyses of cumulative HLC that do not account for changes in the time pattern of HLC counts due to the intervention. However, they are exposed to a high dosage of transfluthrin in the SFS and likely get disarmed or killed, resulting in underestimating repellency and overestimating killing/disarming. The semi-field experiments were designed to keep mosquitoes host seeking, in particular by releasing starved mosquitoes and by removing all opportunity for sugar feeding from the semi-field site, which might prevent mosquitoes from being disarmed and therefore correct partly for this misestimation.

Therefore, field studies are required to confirm the community-level impact and provide more accurate estimates than these current estimates from the semi-field studies. Further studies are also needed to answer the important question on how spatial repellents interact with ITNs, since a strong spatial repellent inhibiting house entry might reduce the killing effect of the ITN.

### Model limitations

As the interventions were designed to target transmission in the peridomestic area in the evening, no HLC was performed between hours 3–11.5 of the experiments, followed by a HLC measure only intended to remove mosquitoes from the screen house. We assumed that the last HLC measure can serve as a proxy for all biting in hours 3–12 since the HLC response was generally very low during hours 3–11.5. Analysing the data only up to hour 3 would likely not change the point estimates of the impact on vectorial capacity, but would increase the corresponding credible intervals.

The HLC data for both the spatial repellent and the push–pull intervention is much more dispersed than the data for the corresponding posterior simulation. This leads to an underestimation of the variability of the effect on vectorial capacity. Allowing the parameters for the repelling and killing/disarming effect to vary by night* via* a hierarchical structure similar to the ones used for the baseline rates may lead to a model that captures this overdispersion. However, we believe that the variability in the relative landing reduction due to the interventions involving transfluthrin-treated eave ribbons can at least partially be explained by a temperature dependence of the corresponding repelling and/or disarming/killing effect.

As HLC is a measure of mosquito landing, and not of biting, we cannot infer a potential biting inhibition effect of the transfluthrin after successful landing. Correspondingly, we make the conservative assumption of no such effect in the vectorial capacity calculation.

The deterministic mosquito model [25] used for our predictions assumes that the number of emerging mosquitoes is independent of the number of adult mosquitoes, which is a valid assumption for settings with high mosquito abundance because of strong density dependence in the larval stages. However, when adult mosquito populations become very small, due to low baseline abundance or because of very strong interventions, this assumption breaks down, and the present methodology would underestimate the effect. At the very least, the results presented here can be seen as a lower bound of the effect size in such situations.

### Estimating community-level impact of other interventions from SFS experiments with HLC data

Our modelling framework can estimate the effect on malaria transmission of any tool acting during the mosquito host-seeking state that is tested in semi-field experiments with time-stratified human landing catch data and some measure on mortality after biting. In particular, the trap can be replaced by any non-human host catching mosquitoes and the spatial repellent by any other product that has a repelling and killing/disarming effect. Of the 21 candidate vector control tools that were included in a systematic review [45], at least the following seven could be tested in semi-field experiments and their transmission effect then be estimated with the present methodology: ‘other attract-and-kill mechanisms’, outdoor effect of ‘eave tubes and eave baffles’, ‘insecticide-treated clothing and blankets’, outdoor effect of ‘insecticide-treated durable wall linings’, ‘insecticide-treated hammocks’, ‘spatial repellents’, ‘topical repellents’. Other potential candidates to be tested in semi-field experiments and analysed with this methodology include ‘insecticide-treated curtains and nets’, ‘insecticide-treated paint’, ‘insecticide-treated plastic sheeting in tents or in temporary shelters’, ‘insecticide-treated tents’ (outdoor effect) and ‘live plants as spatial repellents’. Our model can also be used to define target product profiles for a given effect size in terms of the presented parameterisation framework. Other entomological endpoints than vectorial capacity, such as EIR, are also possible.

It is straightforward to adapt a different HLC time scheme; however, it is important to have time-stratified HLC counts over the time of the experiment with the highest HLC response. For modelling purposes, we recommend running preliminary semi-field experiments with 15-min intervals for HLC counts and to then choose four to ten consecutive HLC periods with approximately equal HLC counts. It is not necessary that the different HLC periods are of the same length, but they need to be consistent over all replicates. Our model can currently use either indoor or outdoor HLC data collected by one volunteer. Experimental designs with volunteers performing HLC first outdoors and then indoors, or with multiple volunteers in the same semi-field compartment are possible after some adjustments of the model. Such experiments are important to understand the differential impact of spatial repellents on indoor and outdoor transmission and to investigate their interaction with indoor tools such as ITNs and IRS. Different interventions at different huts in the same semi-field compartment are also possible by estimating deterrency effects per house.

The effect on malaria incidence and mortality of any intervention parameterised from SFS experiments with time-stratified HLC by the presented methodology can be simulated with models of malaria in humans, such as the platform OpenMalaria, for a variety of different settings.

## Conclusions

This analysis of semi-field data suggests, under the assumption of generalisability to a field situation, that transfluthrin-treated eave ribbons are a promising tool against malaria transmission by *An. arabiensis* in the peridomestic area by providing both personal and community protection. We found that this tool inhibits mosquito landing to a large extent by killing or disarming (suppressing host-seeking behaviour for at least 1 day) and not only repelling mosquitoes, making the combination with traps less necessary in order to protect non-users.

The novel methodology presented here allows us to differentiate between the repelling effect on one hand and the killing or disarming effect on the other hand of outdoor interventions tested in semi-field experiments from time-stratified HLC counts only. With this methodology a potential increase of the risk for non-users after deployment of spatial repellents with incomplete coverage can be quantified. We highlight the need for amended semi-field experiments to detect disarming, as this cannot be distinguished from killing using the present data. Our methodology enables estimates of the impact of the intervention on vectorial capacity, accounting for personal as well as community protection under some assumptions and including uncertainty intervals. This knowledge is important for assessing the effectiveness of candidate tools in an early stage of product development in order to focus on the right tools to fight outdoor malaria transmission.

## Supplementary information

**Additional file 1: Appendix.** A detailed elaboration of the continuous-time Markov chain model, the non-central version of the hierarchical Bayesian model for semi-field experiments over multiple nights, the detailed parameter inference for both the semi-field system and the delayed mortality model and additional results on the inhibition of host-seeking behaviour for multiple days.

**Additional file 2: S1.** The Stan code for the semi-field system model for a single experimental arm (control or intervention) which does not involve traps.

**Additional file 3: S2.** The Stan code for the semi-field system model for a single experimental arm (control or intervention) which involves traps.

**Additional file 4: S3.** The Stan code for the semi-field system model including both the spatial repellent intervention and the control arm.

**Additional file 5: S4.** The Stan code for the semi-field system model including both the trap intervention and the control arm.

**Additional file 6: S5.** The Stan code for the semi-field system model including both the push-pull intervention and the control arm.

**Additional file 7: S6.** The Stan code for the delayed mortality model for a single experimental arm (control or intervention).

**Additional file 8: S7.** The Stan code for the delayed mortality model for both the intervention and the control arm in order to estimate the killing effect after biting.

## Data Availability

The Stan model code is available as additional information.

## Abbreviations

*An.*: Anopheles
HLC: Human landing catch
ITN: Insecticide treated Net
IRS: Indoor residual spraying
R: Repellent
T: Trap
P: Push–pull
C: Control
I: Intervention
MB5: Mbita blend 5
SFS: Semi-field system
