# Supplementary material for: Predicting the impact of outdoor vector control interventions on malaria transmission intensity from semi-field studies
Source: Parasit Vectors. 2021 Jan 20;14:64. doi: 10.1186/s13071-020-04560-x (PMC7819244; doi:10.1186/s13071-020-04560-x)
Supplement: Supplementary file 8 — Additional file 8: S7. The Stan code for the delayed mortality model for both the intervention and the control arm in order to estimate the killing effect after biting. [file 13071_2020_4560_MOESM8_ESM.pdf]

Appendix to ‘Predicting the impact of outdoor vector control interventions on malaria transmission intensity from semi-field studies’

Adrian Denz[1,2], Margaret M. Njoroge[3,4], Mgeni M. Tambwe[5], Clara Champagne[1,2], Fredros Okumu[5,7,8,9], Joop J.A. van Loon[4], Alexandra Hiscox[4,6], Adam Saddler[1,2,5], Ulrike Fillinger[3], Sarah J. Moore[1,2,5], and Nakul Chitnis[1,2]

[1]*Swiss Tropical and Public Health Institute, Department of Epidemiology and Public Health, 4051, Basel, Switzerland*
[2]*University of Basel, Petersplatz 1, Basel, Switzerland*
[3]*International Centre of Insect Physiology and Ecology, Human Health Theme, 00100 Nairobi, Kenya*
[4]*Wageningen University, Laboratory of Entomology, P.O. Box 16, 6700 AA, Wageningen, The Netherlands*
[5]*Ifakara Health Institute, Environmental Health and Ecological Sciences Department, P. O. Box 53, Ifakara, Tanzania*
[6]*London School of Hygiene & Tropical Medicine, ARCTEC, Keppel Street, WC1E 7HT, London, United Kingdom*
[7]*University of the Witwatersrand, Faculty of Health Science, School of Public Health, Johannesburg, South Africa*
[8]*Nelson Mandela African Institution of Science and Technology, School of Life Science and Biotechnology, P. O. Box 447, Arusha, Tanzania*
[9]*University of Glasgow, Institute of Biodiversity, Animal Health and Comparative Medicine, Glasgow, G128QQ, United Kingdom*

Contents

A Elaboration of Continuous Markov chain model for host seeking behaviour **3**

B Non-central version of Hierarchical Bayesian model for semi-field experiments over multiple nights **3**

C Parameter inference for semi-field model **4**
C.1 Intermediate, night-unspecific parameters for semi-field model . . 4
C.2 Rates for semi-field experiments 8
C.3 Normalised nightly variation in semi-field experiments 9
C.4 Nightly variation of rates in semi-field experiments 9
C.5 Parameter correlation in semi-field model 11

D Parameter inference for delayed mortality and postprandial killing effect **13**
D.1 Independent, night-unspecific parameters for delayed mortality model 13
D.2 probability for delayed death 13
D.3 Normalised nightly variation in delayed mortality model 13
D.4 Nightly variation of probabilities in delayed mortality model . . . 18
D.5 Parameter correlation in semi-field model 18

E Inhibition of host-seeking behaviour for multiple days **18**

A Elaboration of Continuous Markov chain model for host seeking behaviour

We assumed that for short times h the probabilities $P_{\mathrm{H}}(h), P_{\mathrm{T}}(h), P_{\mathrm{M}}(h)$ can be approximated linearly in time with constant rates α_{H_k}, α_{T_k} and μ_k, respectively, and that P_{A} is the complementary probability of the sum of the other probabilities. This uniquely defines a a time-homogeneous, continuous-time Markov chain $X(t)$ on the finite state space $\{A, H, T, M\}$ ([1], Theorem 5.2.7, or [2], section VI.6). $X(t)$ is characterised by the infinitesimal generator matrix

$$\mathbf{Q} = \begin{bmatrix} -(\alpha_{\mathrm{H}_k} + \alpha_{\mathrm{T}_k} + \mu_k) & \alpha_{\mathrm{H}_k} & \alpha_{\mathrm{T}_k} & \mu_k \\ 0 & 0 & 0 & 0 \\ 0 & 0 & 0 & 0 \\ 0 & 0 & 0 & 0 \end{bmatrix} \tag{A.1}$$

and the initial probability distribution $P[X(0) = A] = 1$. The transition probability function $\mathbf{P}(t) = (P_{i,j}(t))_{i,j}$ is a matrix giving the probability $P_{i,j}(t)$ of a transition from state i to j after time t for any $i, j \in \{\mathrm{A}, \mathrm{H}, \mathrm{T}, \mathrm{M}\}$. The transition probability function for our model satisfies

$$\tfrac{d\mathbf{P}}{dt}(t) = \mathbf{QP}(t) \tag{A.2}$$

with initial condition that $\mathbf{P}(0)$ is the identity matrix. With the solution formula $\mathbf{P}(t) = \exp(\mathbf{Q}t)$ one finds the transition probability function

$$\mathbf{P}(t) = \begin{bmatrix} P_{\mathrm{A}}(t) & P_{\mathrm{H}}(t) & P_{\mathrm{T}}(t) & P_{\mathrm{M}}(t) \\ 0 & 1 & 0 & 0 \\ 0 & 0 & 1 & 0 \\ 0 & 0 & 0 & 1 \end{bmatrix}, \tag{A.3}$$

with probabilities

$$\begin{aligned} P_{\mathrm{A}}(t) &= e^{-(\alpha_{\mathrm{H}_k} + \alpha_{\mathrm{T}_k} + \mu_k)t)} \\ P_{\mathrm{H}}(t) &= (1 - e^{-(\alpha_{\mathrm{H}_k} + \alpha_{\mathrm{T}_k} + \mu_k)t)}) \tfrac{\alpha_{\mathrm{H}_k}}{\alpha_{\mathrm{H}_k} + \alpha_{\mathrm{T}_k} + \mu_k} \\ P_{\mathrm{T}}(t) &= (1 - e^{-(\alpha_{\mathrm{H}_k} + \alpha_{\mathrm{T}_k} + \mu_k)t)}) \tfrac{\alpha_{\mathrm{T}_k}}{\alpha_{\mathrm{H}_k} + \alpha_{\mathrm{T}_k} + \mu_k} \\ P_{\mathrm{M}}(t) &= (1 - e^{-(\alpha_{\mathrm{H}_k} + \alpha_{\mathrm{T}_k} + \mu_k)t)}) \tfrac{\mu_k}{\alpha_{\mathrm{H}_k} + \alpha_{\mathrm{T}_k} + \mu_k}, \end{aligned} \tag{A.4}$$

for staying in 'A', moving from 'A' to 'H', moving from 'A' to 'T' and moving from 'A' to 'M', respectively, within time t.

B Non-central version of Hierarchical Bayesian model for semi-field experiments over multiple nights

We explicitly give the parameterisation and equations for the non-central version of Hierarchical Bayesian model for semi-field experiments over multiple nights.

symbol	description
C_R	identifier for control arm in spatial repellent experiment
I_R	identifier for intervention arm in spatial repellent experiment
C_T	identifier for control arm in trap experiment
I_T	identifier for intervention arm in trap experiment
C_P	identifier for control arm in push-pull experiment
I_P	identifier for intervention arm in push-pull experiment

Table 1: Notation for experimental arms.

To avoid parameter correlation originating from the hierarchical model structure [3], we transform (9) to the non-central equivalent

$$\begin{aligned} \alpha_{\mathrm{H}_k} &= \exp(a + \phi_k \sigma_a) \\ \alpha_{\mathrm{T}_k} &= \exp(b + \eta_k \sigma_b) \\ \mu_k &= \exp(m + \psi_k \sigma_m), \end{aligned} \tag{B.5}$$

where

$$\phi_k,\, \psi_k,\, \eta_k \sim \mathcal{N}(0,1) \tag{B.6}$$

for all $k \in \{1, ..., 16\}$. Equation (11) still holds; equation (10) then becomes

$$\begin{aligned} &\mathrm{pr}[a, b, m, \sigma_a, \sigma_b, \sigma_m, \phi_1, ..., \phi_{16}, \eta_1, ..., \eta_{16}, \psi_1, ..., \psi_{16} | D_1, ..., D_{16}] \\ &\quad = \sum_{k=1}^{16} \left\{ l_1(\alpha_{\mathrm{H}_k}, \alpha_{\mathrm{T}_k}, \mu_k | D_k) + \mathrm{pr}[\phi_k] + \mathrm{pr}[\eta_k] + \mathrm{pr}[\psi_k] \right\} \\ &\qquad + \mathrm{pr}[a, b, m, \sigma_a, \sigma_b, \sigma_m] - c \end{aligned} \tag{B.7}$$

and equation (12) becomes

$$\begin{aligned} &\mathrm{pr}[\pi, \kappa, \rho, a, m, \sigma_a, \sigma_m, \phi_1, ..., \phi_{16}, \psi_1, ..., \psi_{16} | D_1[\mathrm{C}], ..., D_{16}[\mathrm{C}], D_1[\mathrm{I}], ..., D_{16}[\mathrm{I}]] \\ &\quad = \sum_{k=1}^{16} \left\{ l_2(\pi, \kappa, \rho, \alpha_{\mathrm{H}_k}, \mu_k | D_k[\mathrm{I}], D_k[\mathrm{C}]) + \mathrm{pr}[\phi_k] + \mathrm{pr}[\psi_k] \right\} \\ &\qquad + \mathrm{pr}[\pi, \kappa, \rho, a, m, \sigma_a, \sigma_m] - c, \end{aligned} \tag{B.8}$$

where $\alpha_{\mathrm{H}_k}, \alpha_{\mathrm{T}_k}, \mu_k$ are given by (B.5) and where $\Pr[\phi_k], \Pr[\eta_k], \Pr[\psi_k]$ are given by (B.6). Hence, the hierarchy is encapsulated in the way each nightly likelihood depends on the former hyperparameters, and all parameters appear to be non-hierarchical.

C Parameter inference for semi-field model

For this section we use the notation presented in Table 1.

C.1 Intermediate, night-unspecific parameters for semi-field model

Figures 1, 2 and 3 show the inference on the location parameters (means) of the rates on the logarithmic scale, once by use of the semi-field model without

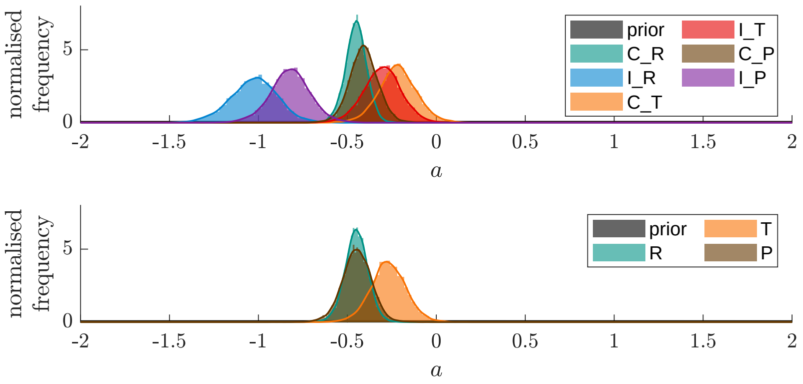

Figure 1: Prior and posterior of a, the mean of the logarithm of the human availability rate

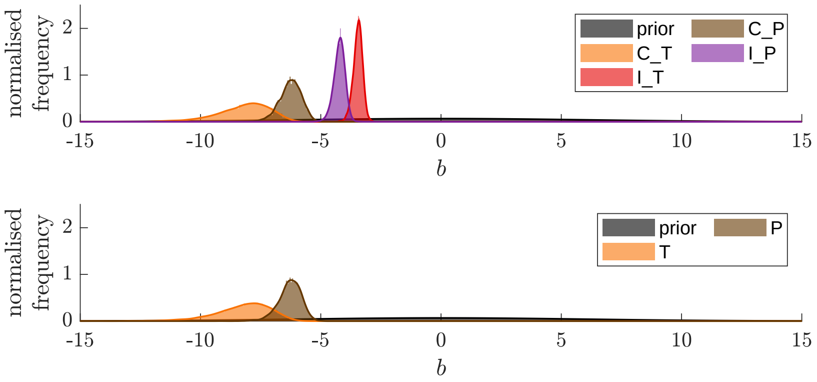

Figure 2: Prior and posterior of b, the mean of the logarithm of the trap availability rate

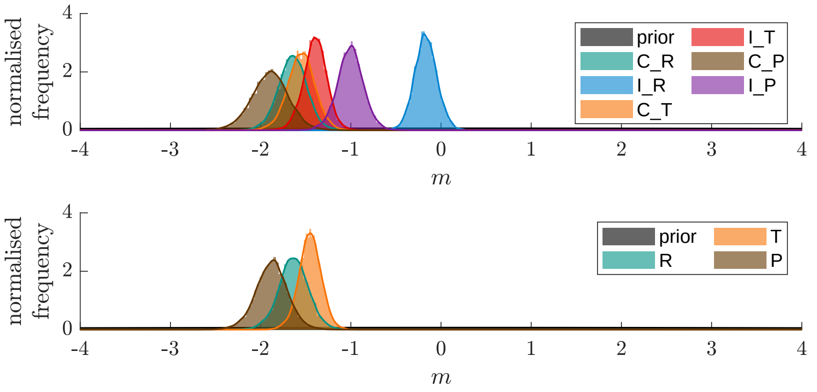

Figure 3: Prior and posterior of m, the mean of the logarithm of the mortality rate

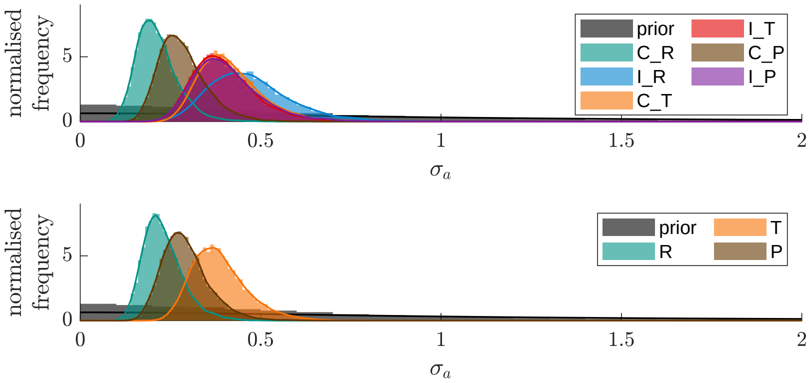

Figure 4: Prior and posterior of σ_a, the standard deviation of the logarithm of the nightly human availability rate

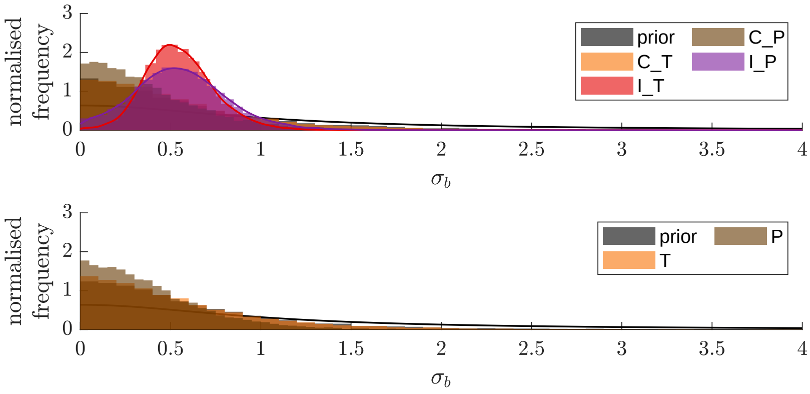

Figure 5: Prior and posterior of σ_b, the standard deviation of the logarithm of the nightly trap availability rate

matching control and intervention (top) and once by use of the model matching them (bottom). Figures 4, 5 and 6 show the inference on the scale parameters (standard deviation) of the rates on the logarithmic scale, once by use of the semi-field model matching control and intervention (top) and once by use of the model without matching them (bottom). Figures 7 and 8 show the inference on the intervention effect parameters by use of the semi-field model matching control and intervention. Running a no-data fit, i.e. replacing all log-likelihood functions that take data as input with 1, should approximately output the priors specified for each parameter if the prior distributions are independent of each other. The no-data fit of the intervention parameters π and ρ did not follow the specified log-normal prior distributions but were much flatter while having the same support. The no-data fit of the scale parameters for the nightly variation, σ_a, σ_b and σ_m, did not follow the specified Half-Cauchy prior distributions, but were more concentrated around 0 while having the same support. This is surprising since the model was written in a non-centered way so that all

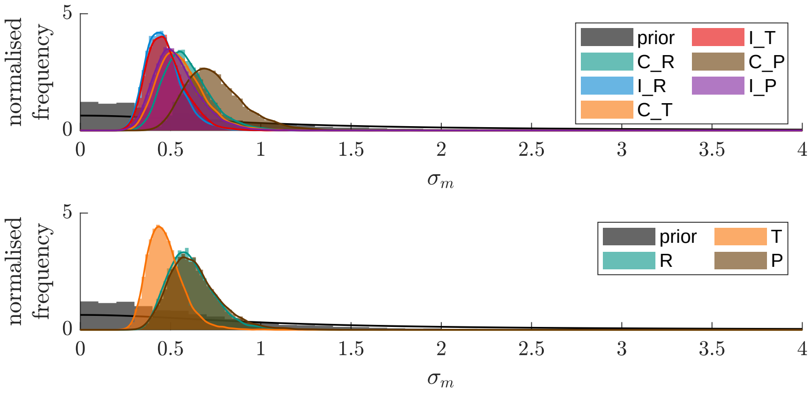

Figure 6: Prior and posterior of σ_m, the standard deviation of the logarithm of the nightly mortality rate

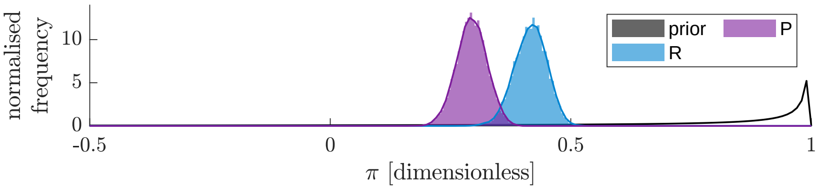

Figure 7: Prior and posterior of π, the relative reduction of the human availability rate

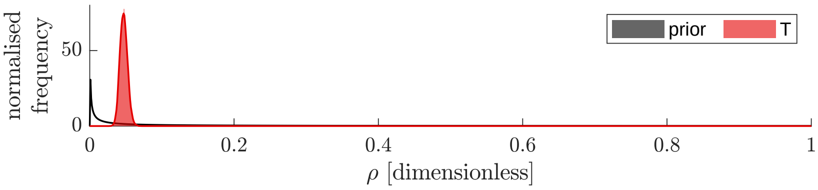

Figure 8: Prior and posterior of ρ, the ratio of the trap availability rate over the human availability rate

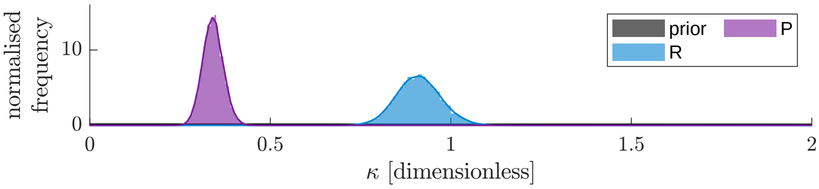

Figure 9: Prior and posterior of κ, the difference of the mosquito mortality rates in treatment and control divided by the control human availability rate

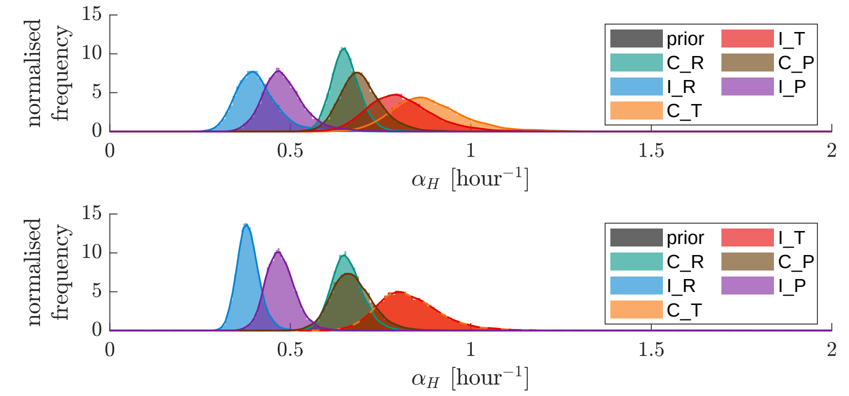

Figure 10: Prior and posterior of the human availability rate α_{H}

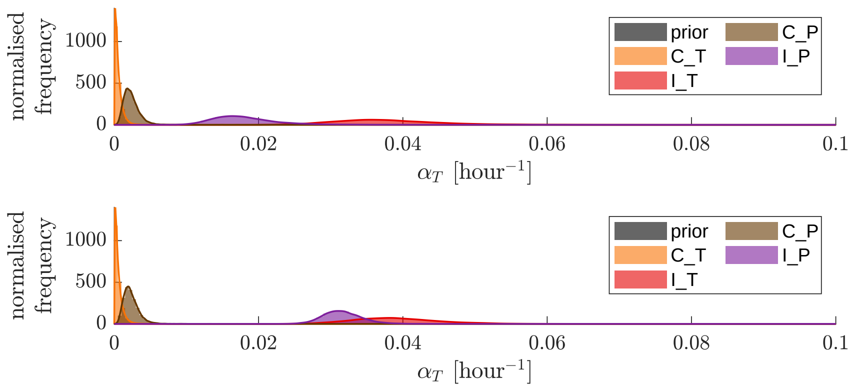

Figure 11: Prior and posterior of the trap availability rate α_{T}

parameters that stan samples are independent. We don't have an explanation for this behaviour. We checked that posteriors were not affected by this by running the model also with much larger scale parameters for the priors. Hence, this has no impact on the parameter inference based on data or on any other result presented in the main text.

C.2 Rates for semi-field experiments

Figures 10, 11 and 12 show the inference on the rates of the semi-field model, which is completely determined by the inference on the intermediate parameters in appendix C.1. The top plot corresponds to the inference by the semi-field model matching control and intervention and the bottom plot corresponds to the inference by the model without matching. Note that Figure 11 is presented without prior in the main text, too, and that the scale is a magnitude smaller than for the other rates. For human availability rate and trap availability rate, the intervention rate depends only on the control rate and an intervention parameter. The posterior of the intervention rate is approximately the posterior of

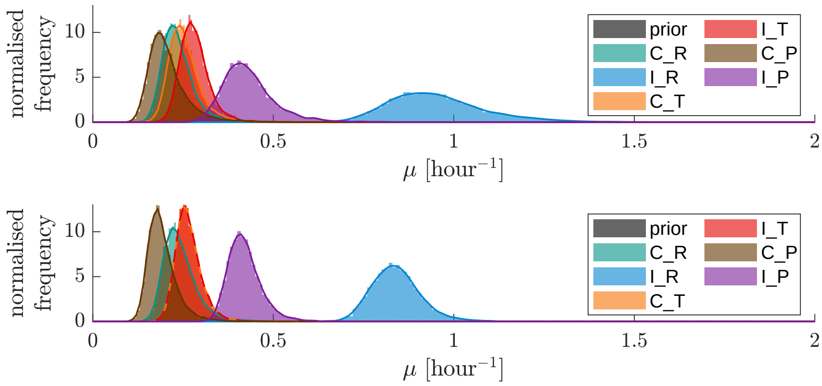

Figure 12: Prior and posterior of the mortality rate μ

the control rate times the posterior of the corresponding intervention parameter if nightly intervention and control rates are positively correlated. It can be seen in figures 16, 17 and 18) that nightly intervention and control rates actually are mostly positively correlated. This is the reason why posteriors from the separate fit (top plot) and matched fit (bottom plot) have very similar means and modes. The dispersion is usually smaller in the matched fit, because of the higher number of data points vs. parameters and because of the nightly correlation of intervention and control rates.

But for mortality, the intervention rate $\mu[\mathrm{I}]$ depends on $\mu[\mathrm{C}]$, κ and $\alpha_{\mathrm{H}}[\mathrm{C}]$ (see equation 6), and if the nightly rates $\mu_k[\mathrm{I}]$ and $\alpha_{\mathrm{H}_k}[\mathrm{C}]$ are not positively correlated, the posterior of $\mu[\mathrm{I}]$ is not the posterior of $\mu[\mathrm{C}]$ plus the posterior of $\alpha_{\mathrm{H}}[\mathrm{C}]$ times the posterior of κ. For the repellent, the posterior of the mortality rate $\mu[\mathrm{I_R}]$ is actually shifted considerably to the left for the matched fit compared to the separate fit.

For the trap experiments, the posteriors for control and treatment human availability rate as well as the posteriors for control and treatment mortality rate lie exactly on top of each other for the matched fit since we parameterise the trap exclusively by the relative availability ρ.

C.3 Normalised nightly variation in semi-field experiments

Figures 13, 14 and 15 show the inference on the parameters capturing the nightly variation (normalised) of the rates on the log-scale in the semi-field model without matching control and intervention for the repellent, trap and push-pull experiments, respectively. Note that the deviation of the log-rates from the mean of the log-rates is normalised here, so neither the magnitude of the deviation from the mean nor the width of the credible intervals can be compared between different rates or different experiments.

C.4 Nightly variation of rates in semi-field experiments

Figures 16, 17 and 18 show the nightly variation of the rates in the semi-field model without matching control and intervention for the repellent, trap and

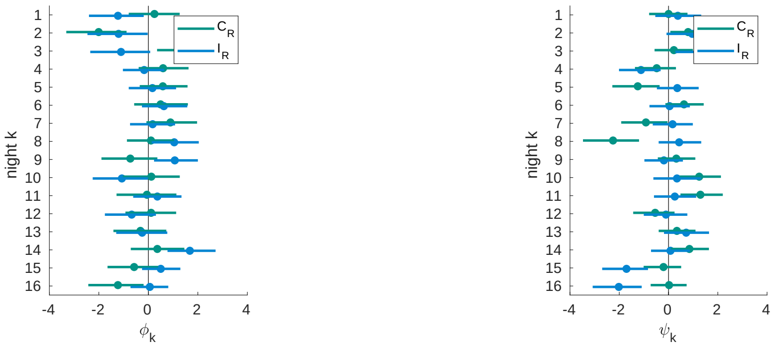

Figure 13: Means and 95%-credible intervals (based on normal approximation) of ϕ_k, the normalised deviation of the logarithms of the human availability rate in each night k from the mean of the logarithm of the human availability rate, and of ψ_k, the normalised deviation of the logarithms of the mortality rate in each night k from the mean of the logarithm of the mortality rate, for control and intervention arm of the repellent experiments.

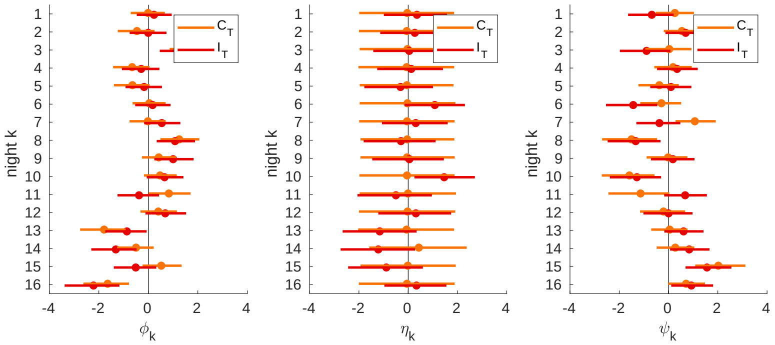

Figure 14: Means and 95%-credible intervals (based on normal approximation) of ϕ_k, the normalised deviation of the logarithms of the human availability rate in each night from the mean of the logarithm of the human availability rate, of η_k, the normalised deviation of the logarithms of the trap availability rate in each night k from the mean of the logarithm of the trap availability rate, and of ψ_k, the normalised deviation of the logarithms of the mortality rate in each night k from the mean of the logarithm of the mortality rate, for control and intervention arm of the trap experiments.

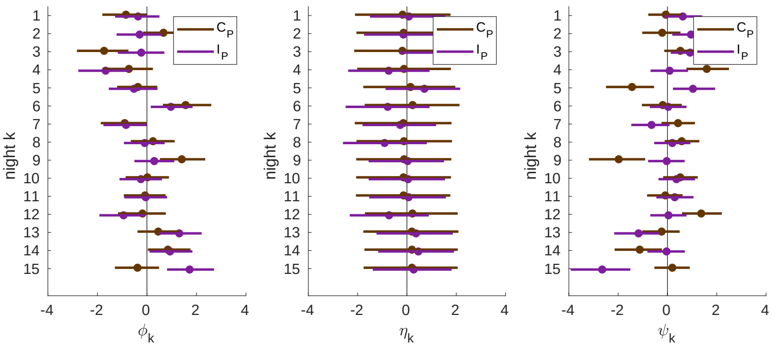

Figure 15: Means and 95%-credible intervals (based on normal approximation) of ϕ_k, the normalised deviation of the logarithms of the human availability rate in each night from the mean of the logarithm of the human availability rate, of η_k, the normalised deviation of the logarithms of the trap availability rate in each night k from the mean of the logarithm of the trap availability rate, and of ψ_k, the normalised deviation of the logarithms of the mortality rate in each night k from the mean of the logarithm of the mortality rate, for control and intervention arm of the push-pull experiments.

push-pull experiments, respectively.

C.5 Parameter correlation in semi-field model

Figures 19, 20 and 21 show the posterior samples of the inferences made with the matched SFS model for repellent only, trap only and push-pull system, respectively, by means of scatter plots of the marginal posterior samples of parameter pairs ('pairs plot'). These plots were generated with the function '`pairs`' in R [4]. These plots are suitable to detect unwanted parameter correlations and identifiability problems. All independent parameters of the model were investigated, but the normalised nightly variations are not shown here. Figures 19 and 21 show negative correlation between the parameters for repelling effect (π) and killing effect (κ). This is not surprising as increasing π and increasing κ both lower the probability to encounter a host, and only the time pattern of the HLC counts over 4 HLC periods can reveal the differential contribution of the two effects. However, this does not constitute an identifiability problem since the correlation is limited to a reasonably small region of the parameter space, i.e. the posterior sample doesn't stretch over the whole parameter space. In Figures 20 and 21 the posterior sample for parameter σ_b is very widespread and seems to be truncated at 0. This is due to the very low number of accidental trap catches in the control experiments, while σ_b is the standard deviation of the log of the corresponding probability.

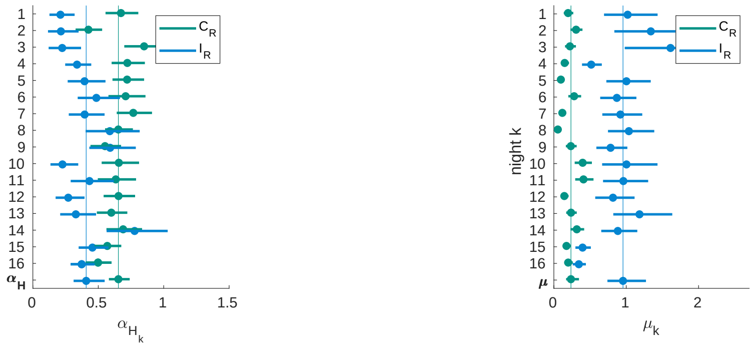

Figure 16: Means and 95%-credible intervals (based on normal approximation) of the human availability rate in each night α_{H_k} and of the mean human availability rate α_{H}, as well as of the mortality rate of each night μ_k and of the mean mortality rate μ, for control and intervention arm of the repellent experiments.

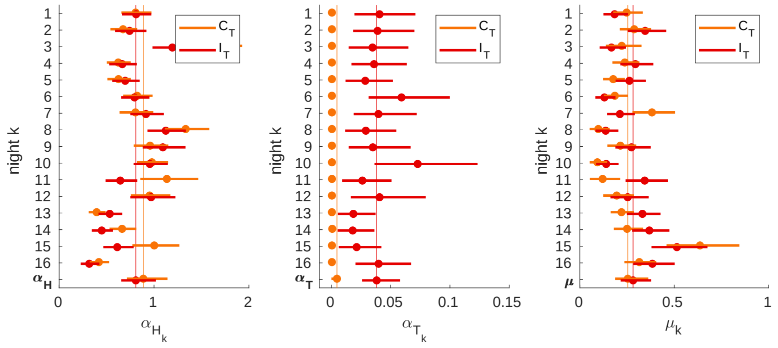

Figure 17: Means and 95%-credible intervals (based on normal approximation) of the human availability rate in each night α_{H_k} and of the mean human availability rate α_{H}, of the trap availability rate in each night α_{T_k} and of the mean trap availability rate α_{T}, as well as of the mortality rate of each night μ_k and of the mean mortality rate μ, for control and intervention arm of the trap experiments.

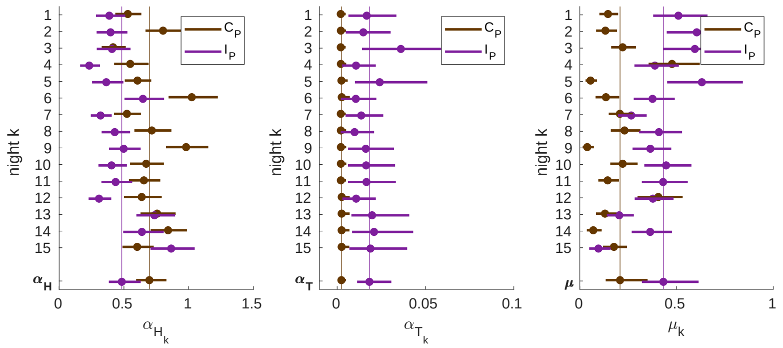

Figure 18: Means and 95%-credible intervals (based on normal approximation) of the human availability rate in each night α_{H_k} and of the mean human availability rate α_{H}, of the trap availability rate in each night α_{T_k} and of the mean trap availability rate α_{T}, as well as of the mortality rate of each night μ_k and of the mean mortality rate μ, for control and intervention arm of the push-pull experiments.

D Parameter inference for delayed mortality and postprandial killing effect

D.1 Independent, night-unspecific parameters for delayed mortality model

Figures 22 shows the inference on the location parameter (mean) of the probability on the logit-scale, once by use of the delayed mortality model matching control and intervention (top) and once by use of the model without matching them (bottom). Figures 23 shows the inference on the scale parameter (mean) of the probability on the logit-scale, once by use of the delayed mortality model matching control and intervention (top) and once by use of the model without matching them (bottom). Figures 24 shows the inference on the postprandial killing effect parameter by use of the semi-field model matching control and intervention.

D.2 probability for delayed death

Figure 25 shows the inference on the rates of the semi-field model, which is completely determined by the inference on the intermediate parameters in appendix D.1. The top plot corresponds to the inference by the semi-field model matching control and intervention and the bottom plot corresponds to the inference by the model without matching.

D.3 Normalised nightly variation in delayed mortality model

Figures 26 shows the inference on the parameters capturing the nightly variation (normalised) of the death probability on the logit-scale in the delayed mortality model without matching control and intervention.

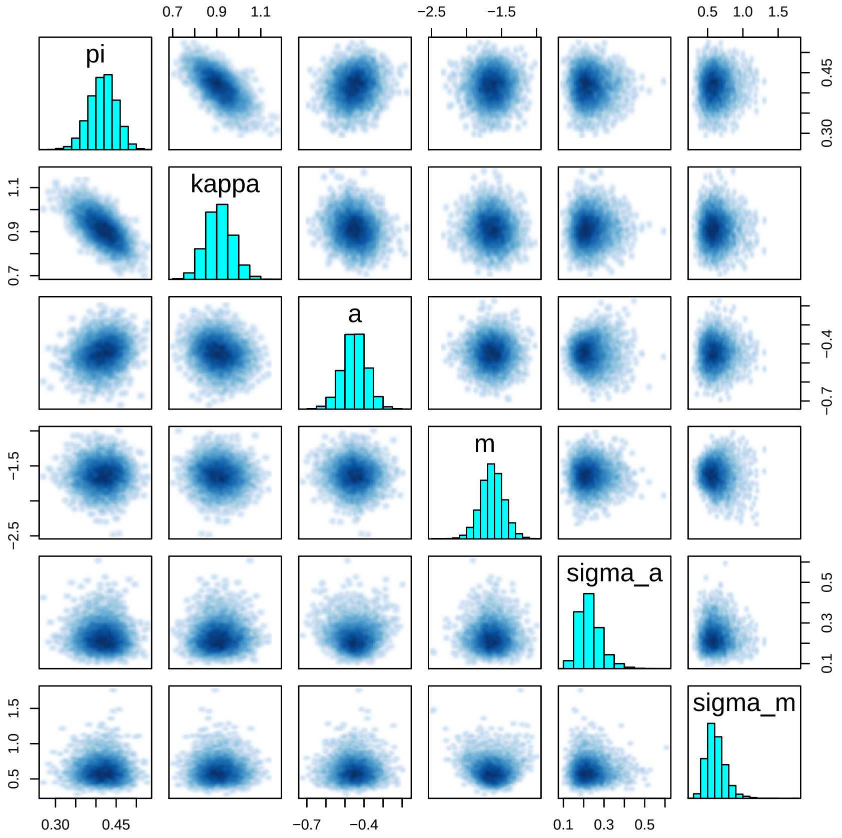

Figure 19: Pairwise scatter plot of the posterior samples of all independent parameters, except the normalised nightly variation, of the matched SFS model fitted to the data of the experiments with repellent only and corresponding control experiments. The diagonal shows the marginal posterior samples of the single parameters.

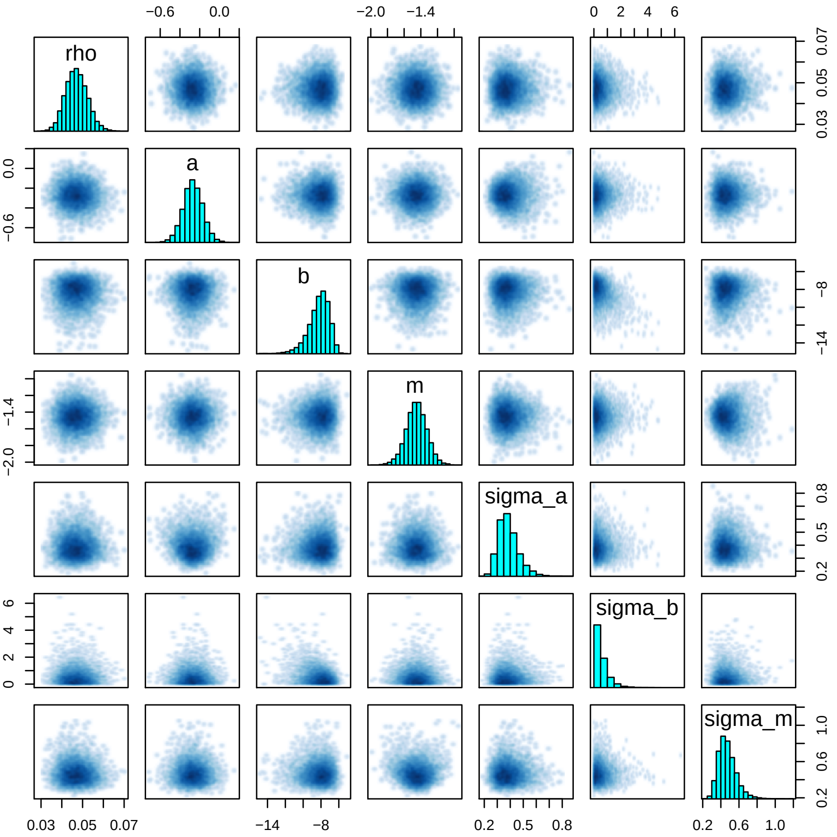

Figure 20: Pairwise scatter plot of the posterior samples of all independent parameters, except the normalised nightly variation, of the matched SFS model fitted to the data of the experiments with trap only and corresponding control experiments. The diagonal shows the marginal posterior samples of the single parameters.

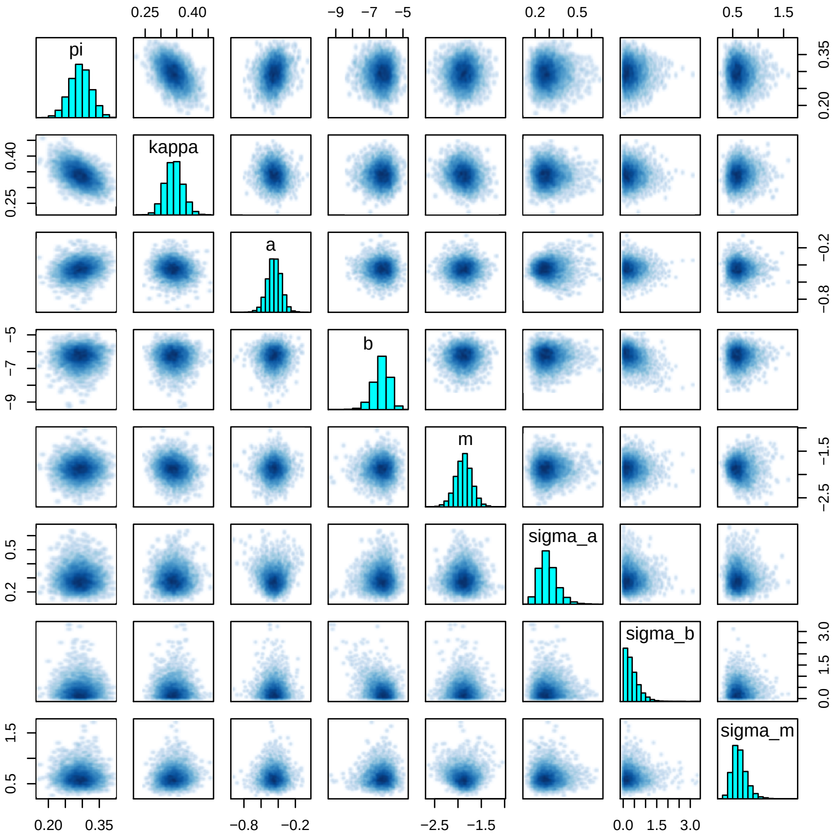

Figure 21: Pairwise scatter plot of the posterior samples of all independent parameters, except the normalised nightly variation, of the matched SFS model fitted to the data of the experiments with the push-pull system and corresponding control experiments. The diagonal shows the marginal posterior samples of the single parameters.

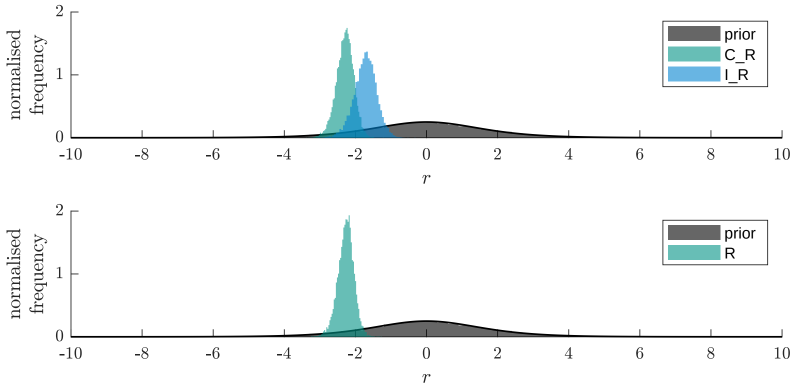

Figure 22: Prior and posterior of r, the mean of the logit of the probability of delayed death

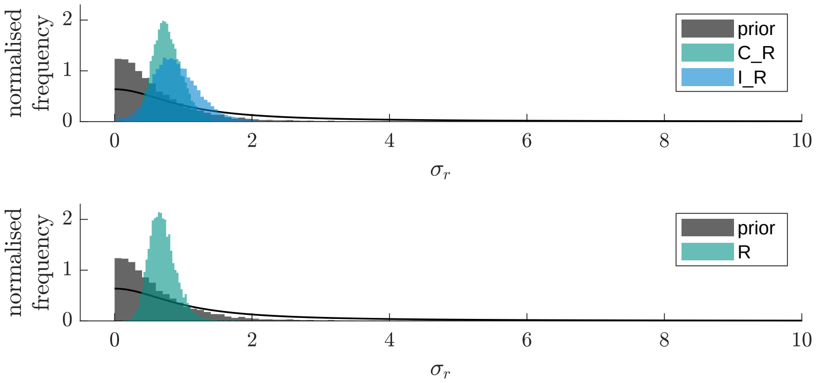

Figure 23: Prior and posterior of σ_r, the standard deviation of the logit of the nightly probability of delayed death

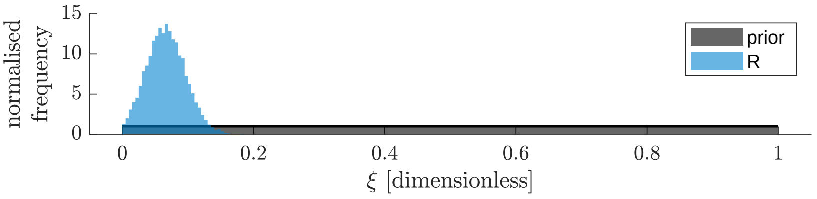

Figure 24: Prior and posterior of the postprandial killing effect ξ

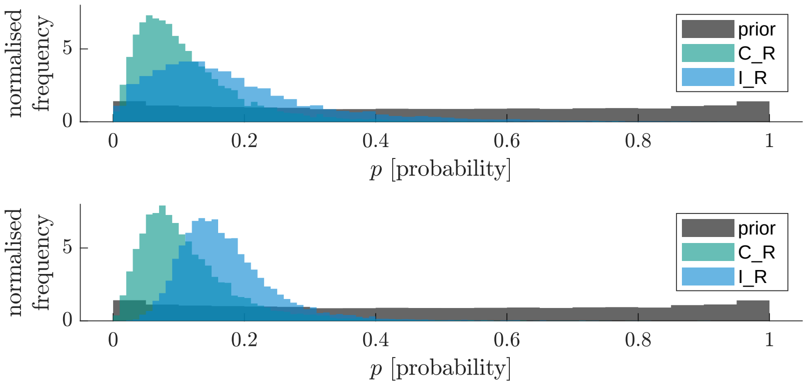

Figure 25: Prior and posterior of the probability of delayed death p

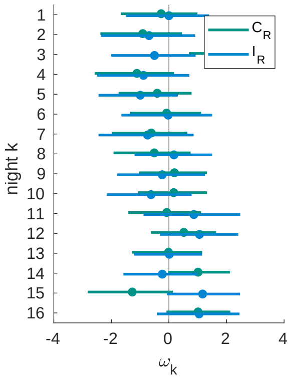

Figure 26: Means and 95%-credible intervals (based on normal approximation) of ω_k, the normalised deviation of the logit of the delayed death probability in each night from the mean of the logit of the delayed death probability, for control and intervention arm of the experiments.

D.4 Nightly variation of probabilities in delayed mortality model

Figures 27 shows the nightly variation of the death probability on the logit-scale in the delayed mortality model without matching control and intervention.

D.5 Parameter correlation in semi-field model

Figure 19, 20 and 21 show the joint posterior samples of the inferences with the matched SFS model for repellent only, trap only and push-pull system, respectively, by means of scatter plots of the marginal posterior samples of pairs of inferred parameters ('pairs plot'). These plots are suitable to detect unwanted parameter correlations and identifiability problems. All independent parameters, except for the normalised nightly variations, are shown, while the latter were also investigated. generated with function '`pairs`' in R [4].

E Inhibition of host-seeking behaviour for multiple days

To model inhibition of host-seeking behaviour for multiple days we send mosquitoes that encountered a shadow host around the feeding cycle without actually feeding, by setting all proportions to 1, except for the proportion P_{C_i} to move to resting state and the proportion P_{D_i} to survive the resting state. Note that this is only possible for a number of days being a multiple of the fixed number of days required to move from state B back to state A with the current model [5].

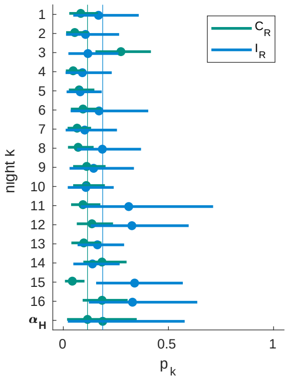

Figure 27: Means and 95%-credible intervals (based on normal approximation) of ω_k, the delayed death probability in each night from the mean of the delayed death probabilities, for control and intervention arm of the experiments.

References

[1] Stroock, D.W.: An Introduction to Markov Processes, 2nd edition edn. Graduate Texts in Mathematics, vol. 230. Springer, New York (2013)

[2] Taylor, H.M., Karlin, S.: An Introduction to Stochastic Modeling, 3rd ed edn. Academic Press, San Diego (1998)

[3] Betancourt, M., Girolami, M.: Hamiltonian Monte Carlo for Hierarchical Models. In: Current Trends in Bayesian Methodology with Applications, 1st edition edn. Chapman and Hall/CRC, New York (2015). doi:10.1201/b18502-11

[4] R Core Team: R: A Language and Environment for Statistical Computing. R Foundation for Statistical Computing. https://www.R-project.org/

[5] Chitnis, N., Smith, T., Steketee, R.: A mathematical model for the dynamics of malaria in mosquitoes feeding on a heterogeneous host population. Journal of Biological Dynamics **2**(3), 259–285 (2008). doi:10.1080/17513750701769857

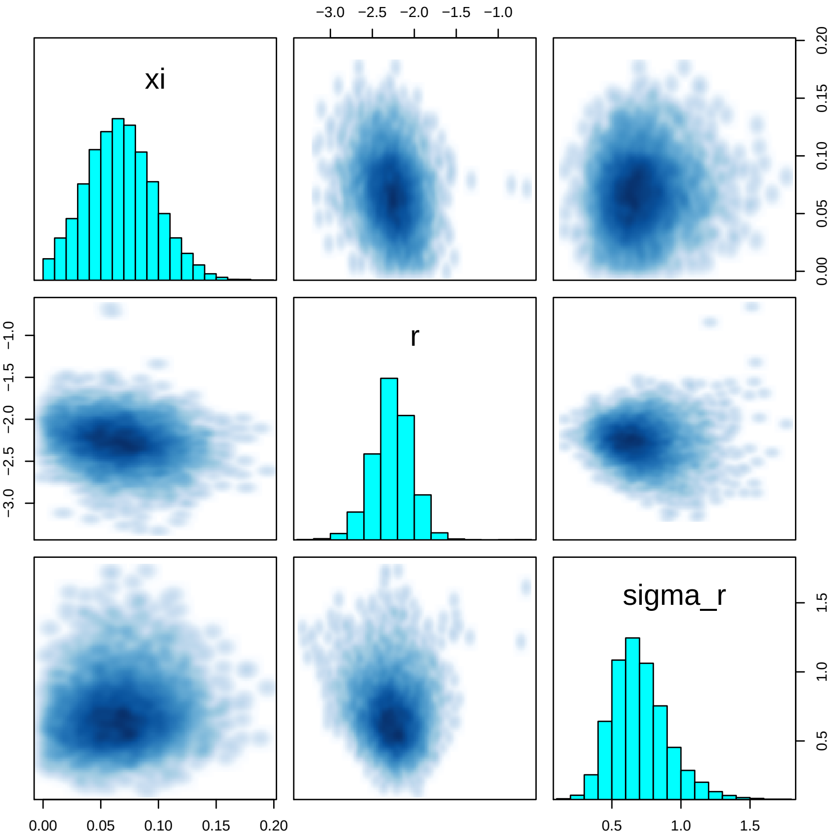

Figure 28: Pairwise scatter plot of the posterior samples of all independent parameters, except the normalised nightly variation, of the matched delayed mortality model fitted to the data of the experiments with repellent only and corresponding control experiments.

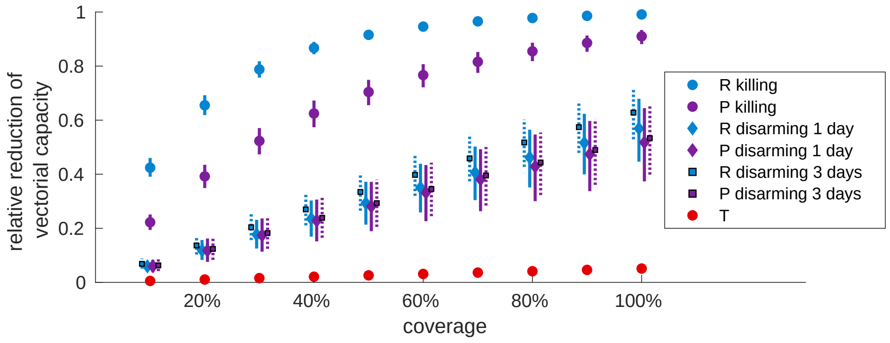

Figure 29
